# Computational deconvolution of drug sensitivity via single-cell–to–bulk transcriptome mapping

**DOI:** 10.1371/journal.pcbi.1014862

**Published:** 2026-10-16

**Authors:** Alexandre Coudray, Romain Forey, Benjamin Bejar Haro, Filipe Martins, Joana Carlevaro-Fita, Shaoline Sheppard, Sandra Eloise Offner, Gioele La Manno, Guillaume Obozinski, Didier Trono

**Affiliations:** 1 EPFL - Ecole Polytechnique Fédérale de Lausanne, Lausanne, Switzerland; 2 SDSC - Swiss Data Science Center, Lausanne, Switzerland; 3 Oncology department, Centre Hospitalier Universitaire Vaudois (CHUV), Hematology Service and Central laboratory, Lausanne, Switzerland; Zhejiang University, CHINA

## Abstract

Analyses routinely conducted in both fundamental research and biomedical studies often report aggregate signals derived from heterogeneous mixtures of cells. These mixtures reflect the intrinsic anatomical and functional complexity of the tissues and organs from which they are derived. Such bulk measurements obscure cell-specific signals, thereby hindering accurate interpretation of the underlying biological processes. Here, we introduce CLIMB and CLIFF, a two-step machine-learning framework that deconvolves bulk RNA-seq into cell-type composition and expression, and then resolves bulk drug sensitivity measurements into cell subtype- and single-cell-specific profiles. We first validate CLIMB using in-vitro mixtures of leukemic cell lines with experimentally known cell-type proportions, then extend this validation to large-scale, cross-dataset pseudo-bulk analyses built from independent scRNA-seq references across two disease contexts and five cancer types — mimicking the reference-to-target mismatches encountered in real applications — and show that CLIMB accurately infers the cellular composition of bulk samples, outperforming state-of-the-art bulk deconvolution methods. We emphasize CLIMB’s effectiveness in acute myeloid leukemia, where it offers highly accurate estimation of leukemic stem cell fractions across three test datasets and three patient cohorts. We then validate CLIFF, our algorithm for estimating cell subtype- and single-cell-specific drug sensitivity, first on the same in-vitro cell line mixtures using experimentally measured drug responses, and then through in-depth, realistic-scale pseudo-bulk simulations. We applied our deconvolution strategy to the beatAML cohort dataset, currently the most extensive database of *ex-vivo* drug screening data. This analysis generated a cell subtype–resolved drug sensitivity landscape for 121 compounds, revealing multiple compounds putatively active against leukemic stem cells, including midostaurin, A-674563, SNS-032, and foretinib. Collectively, our work introduces a general computational framework for resolving bulk functional measurements at cell-type and single-cell resolution, providing an attractive new computational tool for drug development and precision medicine.

## Author summary

Cells can be taken from patients and used to directly test panels of drugs, offering personalized guidance for therapeutic decisions based on the patient’s own cells. These samples are often composed of mixtures of cell types, so the observed signal reflects only the average response over all cells, making it difficult to determine which populations are sensitive or resistant to treatment. Although new technologies can assess RNA expression in individual cells and reveal distinct populations, linking this information to drug response measurements remains challenging. In this study, we introduce a computational approach that helps bridge this gap. By combining gene expression data with drug sensitivity measurements, our method estimates both the cellular composition of samples and how different cell populations respond to drugs. We demonstrate that this approach accurately identifies cell-type composition across multiple cancers and diseases. Applying our framework to acute myeloid leukemia, we analyzed the largest available dataset of patient drug screening experiments and uncovered novel drug response patterns, including compounds predicted to target leukemic stem cells, a cancer cell type capable of initiating disease relapse. This work provides a way to dissect drug response data at the level of individual cell types, revealing hidden patterns and potentially enabling more precise, targeted treatment strategies.

## Introduction

Deciphering drug sensitivity in heterogeneous cell populations remains a central challenge in functional genomics, as most experimental assays only capture signals at the bulk level from mixed cell populations. This limitation is illustrated by *ex-vivo* drug sensitivity screening (DSS), which enables parallel testing of hundreds of compounds on highly heterogeneous, freshly isolated tumor cells [1–3]. However, DSS measures drug responses at the level of mixed cell populations rather than individual cell subtypes. Coupling *ex-vivo* DSS with cell purification techniques such as fluorescence-activated cell sorting (FACS) can partly address this shortcoming, but sorting requires large quantities of cells, is limited by the availability of specific cell surface markers, and the procedure itself compromises cell integrity [4]. Therefore, computational methods capable of deconvoluting *ex-vivo* DSS would constitute attractive alternatives, as they would alleviate the need for cell subtypes purification. More broadly, many functional assays in biomedicine - including cell viability assays, perturbation screens, and metabolic activity assays - are conducted at the bulk level. Computational deconvolution approaches have been successfully applied to bulk transcriptomic data primarily to infer cell-type composition [5–9] by leveraging cellular states characterized by single-cell transcriptomics, but extending these concepts to bulk functional readouts such as drug sensitivity remains largely unexplored.

Acute myeloid leukemia (AML) provides a particularly well-suited disease context for the application of deconvolution methods, owing to its pronounced cellular heterogeneity and the strong association between differentiation state and drug response [10–12]. A persistent challenge in AML is drug resistance and relapse, urging a deeper grasp of the biological factors underpinning drug response. Notably, drug response frequently aligns with tumor cell maturation state, with certain drugs effective against less differentiated states [13–19], and others against more differentiated states [13,16,17,20–27]. Recent single-cell sequencing further delineated AML cancer cell subtypes tied to differentiation state, including hematopoietic-stem-cell-like, progenitor-like, GMP-like, promonocyte-like, monocyte-like, and cDC-like types [28], several of which are directly relevant to the oncogenic process. Leukemic stem cells (LSCs) represent the relapse-competent fraction of the HSC-like cancer cell subtype identified by Van Galen et al. and are driving relapse and reduced survival in AML patients [29–32]. Gene expression signatures from these leukemic stem cells (LSCs) also offer valuable prognostic insights [33–35], underscoring their clinical importance. Given this context, we posit that innovative approaches to elucidate the drug sensitivity of distinct cancer cell subpopulations could significantly enhance AML treatment strategies and potentially offer new avenues for targeting LSCs.

In this work, we introduce novel computational techniques that enable the concurrent estimation of cell subtype proportions and the deconvolution of *ex-vivo* drug sensitivity screening data into cell subtype- or single-cell-specific drug sensitivity profiles. We first developed CLIMB, a tool designed to decipher the cell subtype composition and expression within bulk samples, using a novel bulk-to-single-cell deconvolution approach. The outcome of CLIMB’s deconvolution serves as direct input for our subsequent method, CLIFF. CLIFF employs a probabilistic model to uncover latent variables corresponding to cell subtype survival probability, and thus deconvolute bulk-level drug sensitivity into cell-subtype-specific drug sensitivity profiles. We benchmarked our methods through the analysis of cancer cell line mixes we generated *in-vitro*, and through pseudo-bulk deconvolution based on eleven single-cell datasets across two disease contexts and five cancer types. We then deployed CLIMB on three cohorts of AML patients, and used CLIFF to deconvolute cell-subtype drug sensitivity based on the beatAML cohort [12]. This led us to predict that the drugs A-674563, midostaurin, SNS-032, and foretinib would be effective in targeting LSCs.

## Results

### Experimental design

Accurate deconvolution of functional readouts requires reliable estimation of cellular composition. We therefore developed CLIMB, a signature matrix–free deconvolution method that infers cell subtype proportions and expression profiles from bulk RNA-seq data using a single-cell RNA-seq reference dataset (Figs 1A, S1, S2). CLIMB models each bulk transcriptome as a non-negative linear combination of individual single-cell profiles, yielding cell-level coefficients that map bulk samples to single cells and leverage the full intercellular variability of the reference dataset. Cell-type proportions are obtained by aggregating these coefficients by annotation, with reference-induced cellular imbalance biases mitigated through an iterative Empirical Bayes sub-sampling procedure. This bulk-to-single-cell mapping further enables the estimation of cell-type–specific expression by combining reference-based expression with learned residual components that capture expression absent from the single-cell reference. As a clinically relevant application, we used CLIMB to estimate leukemic stem cell fractions in bulk samples from patients with acute myeloid leukemia.

**Fig 1:**
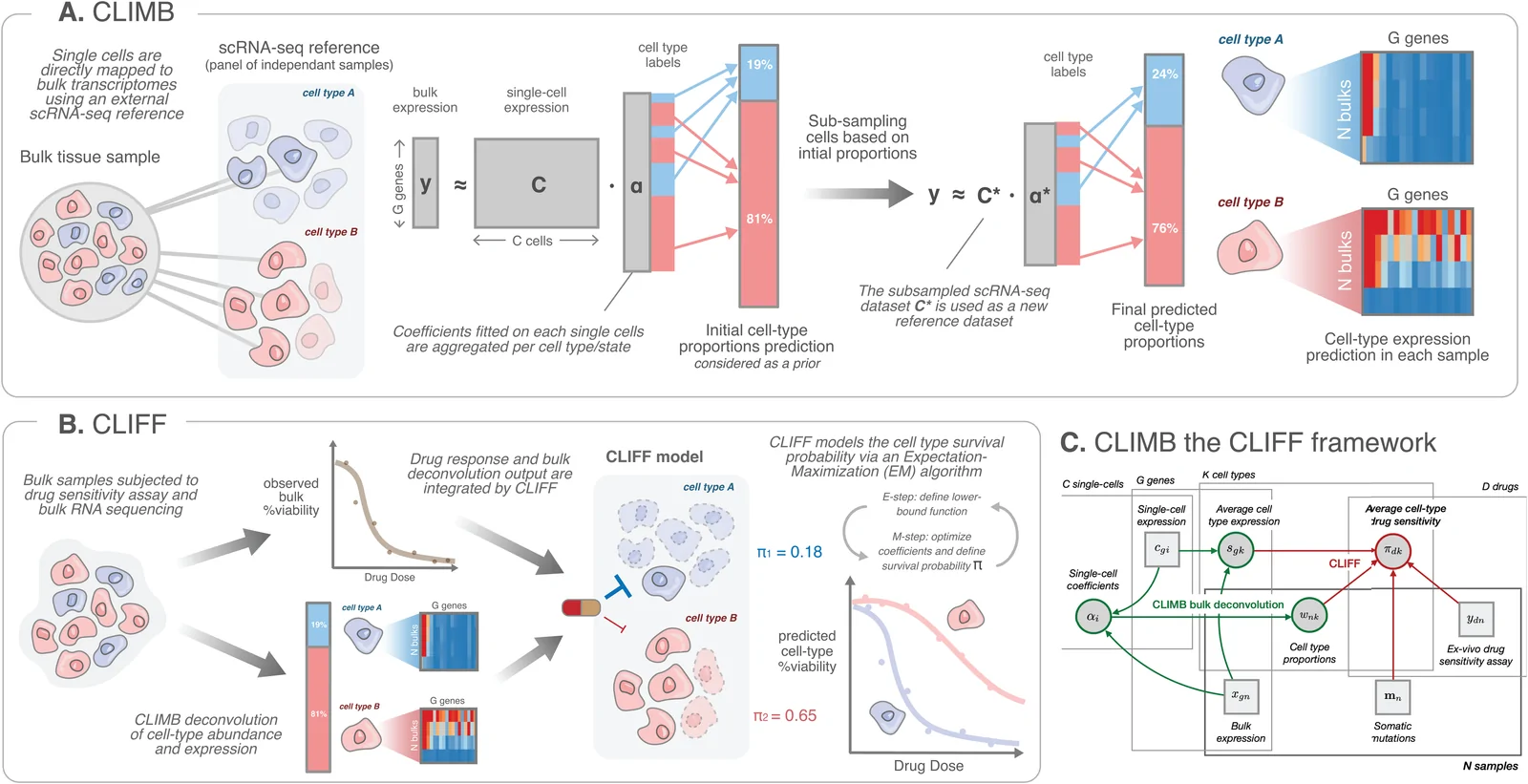
Experimental Design. **(A) Overview of CLIMB.** CLIMB deconvolves bulk RNA-seq data by mapping each bulk transcriptome to individual single cells from a reference scRNA-seq dataset, using iterative Empirical Bayes sub-sampling to reduce reference bias, and enables estimation of cell subtype proportions and cell-type–specific expression. **(B) Overview of CLIFF.** Building on CLIMB output, CLIFF is a probabilistic model that infers cell-type–specific drug sensitivities from bulk survival measurements by modeling observed responses as weighted mixtures of latent cell-subtype survival probabilities, with parameters estimated via an Expectation–Maximization algorithm. **(C) Framework overview.** Schematic of the two-step CLIMB–CLIFF framework, illustrating the links between input data, learned variables, and outputs across both methods.

Building on the output of CLIMB, we developed CLIFF, a probabilistic model that infers cell-type–specific drug sensitivities from bulk drug response measurements by modeling observed survival rates as weighted mixtures of latent cell-subtype survival probabilities, using known cell-type proportions (Figs 1B, S1). Cell-subtype drug sensitivity is linked to gene expression through a sigmoid model, and parameters are estimated via an Expectation–Maximization algorithm. By integrating CLIMB-derived single-cell coefficients, CLIFF can be naturally extended to single-cell resolution (CLIFF-SC), enabling inference of drug sensitivity at the level of individual cells without requiring direct single-cell perturbation data. The framework can optionally incorporate somatic mutations as covariates and supports a high-resolution mode in which drug sensitivity is estimated within each bulk sample (S1B Fig).

This framework was evaluated using both experimental and computational strategies. First, we generated mixtures of human leukemic cell lines, providing a controlled system with known cell proportions and experimentally measurable cell line–specific drug sensitivities. Second, we applied CLIMB and CLIFF to bulk RNA-seq and *ex-vivo* drug sensitivity data from the beatAML cohort, enabling translation of bulk drug response measurements into cell subtype–resolved profiles.

### Evaluating bulk deconvolution methods for predicting cellular composition in cancer cell line mixtures

CLIMB infers cell-subtype proportions and can thus predict the composition of a cell mix (Fig 2A). To test its efficiency and compare it with published methods, we generated *in-vitro* pools containing various ratios of HL60, SUDHL4, K562, and THP1 human leukemic cell lines. We additionally performed single-cell RNA sequencing of an equimolar mixture of the four cell lines and used this dataset as a reference for all deconvolution methods, applying the same cell and gene selection criteria (employing genes with higher estimated biological variance than technical variance; see Methods). We could thus use these pre-determined ratios as ground truth to compare the predictive value of CLIMB (Fig 2B, 2C) and a panel of seven bulk deconvolution methods: BayesPrism [5], CiberSortX [6], TAPE [7], Scaden [8], MuSiC [9], BisqueRNA [36], and NNLS [9], using random proportions as a negative control. The overall ranking across four performance metrics–-root mean square error (RMSE; lower values indicate better performance), Pearson correlation coefficient (PCC), Spearman correlation coefficient (SCC), and coefficient of determination (R-square)–-was computed by averaging the ranks obtained for each metric (mean ± standard deviation). This analysis identified CLIMB and Scaden as the top-performing deconvolution methods (Fig 2D), followed by BayesPrism. Notably, CLIMB reached a higher accuracy than other deconvolution methods based on the root mean square error (RMSE, Fig 2E). Scaden reached similar PCC, SCC, and R-squared, than CLIMB (Fig 2C–2F). A wilcoxon test taking four metrics into account showed significant advantage of CLIMB over other deconvolution methods, except Scaden. In summary, CLIMB demonstrated top-ranking accuracy in deconvoluting bulk RNA-seq samples from *in-vitro* cell mixtures, matching the performance of the deep learning-based method Scaden and surpassing other deconvolution methods.

**Fig 2:**
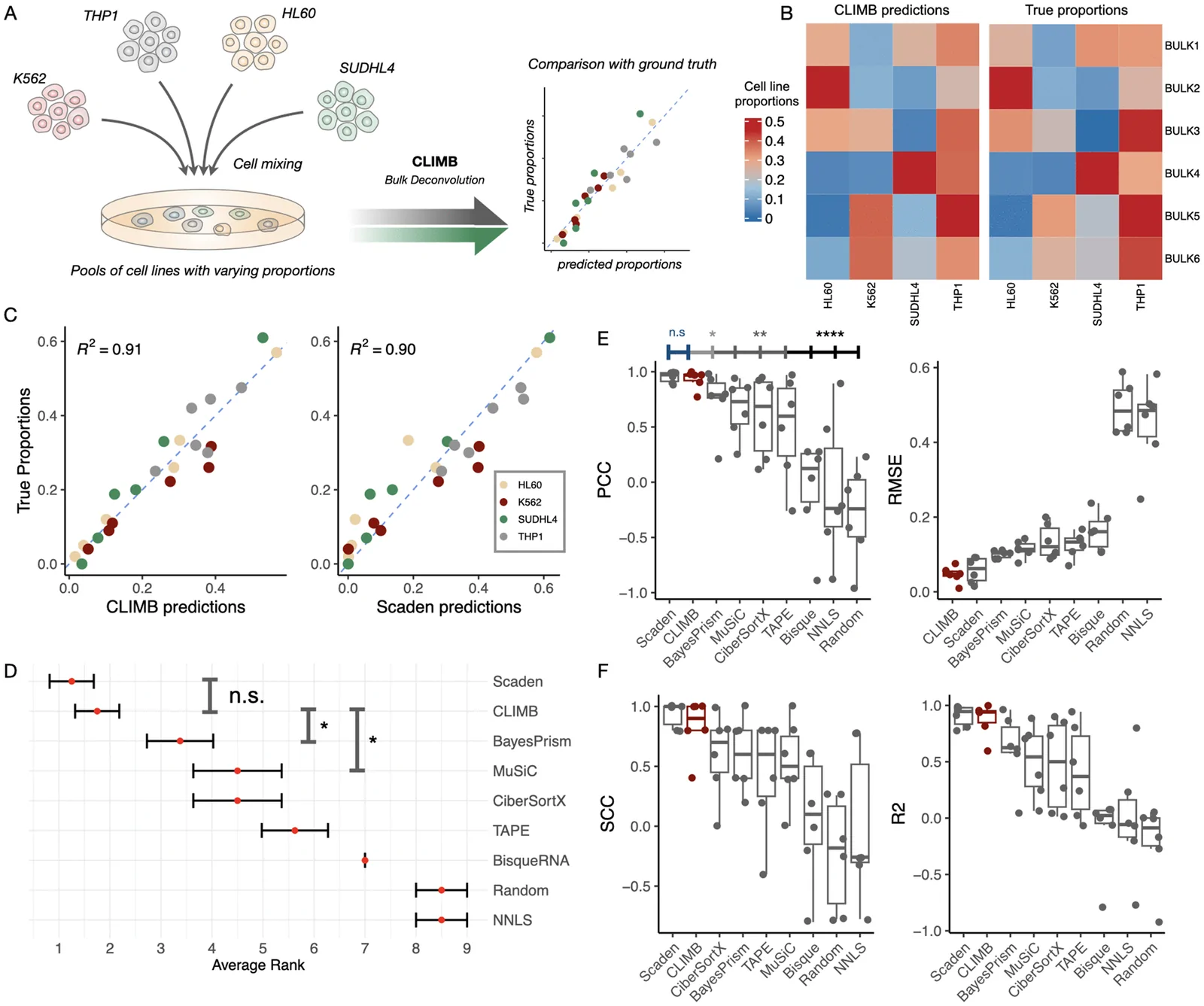
Cell Line Abundance Deconvolution using an *In-Vitro* Experiment with Known Proportions. **(A)** Experimental design for *in-vitro* experiment involving mixtures of human leukemic cell lines. **(B)** Heatmap displaying predicted cell line proportions by CLIMB (left) and actual proportions (ground truth, right). **(C)** Scatter plot comparing true versus predicted cell line abundances using CLIMB (left) and Scaden (right), with colors representing cell lines (6 bulk samples and 4 cell lines). **(D)** Overall results ranked based on median of four metrics (PCC, SCC, RMSE, and R-square) for bulk sample-level comparison. Wilcoxon rank test used for statistical comparison between CLIMB and other methods. **(E)** Left: Comparison of deconvolution accuracy using Pearson correlation coefficient (PCC) between predicted and true cell line proportions. Statistical significance assessed using Pearson and Filon method with *cocor* R package. Left boxplot depicts PCC *per sample* (4 true versus 4 predicted cell line abundances for 6 bulk samples). Right: same approach using Spearman Correlation Coefficient. **(F)** Left: Similar to (**E**) but for R^2^ metric. Right: similar to (**E**) but for root mean square error (RMSE) metric.

### Evaluating non-negative least squares performance relative to CLIMB in bulk and pseudo-bulk settings

Although non-negative least squares (NNLS) is expected to perform well for deconvolution, it did not outperform random proportions in the *in-vitro* cell mixture experiments (Fig 2). To investigate this unexpected result, we generated six pseudo-bulk samples from the same scRNA-seq reference dataset, using exactly the same cell proportions as the six real bulk RNA-seq samples obtained from experimental cell mixes. Half of the cells were used to construct the pseudo-bulk samples, while the remaining cells were retained as the reference. Deconvolution of these pseudo-bulk samples resulted in high accuracy (>95%) for CLIMB and for two independent NNLS implementations (from MuSiC library or with a custom NNLS implementation based on average cell-type expression), with CLIMB performing marginally better (S9A Fig). In contrast, when deconvolving real bulk RNA-seq samples using the same scRNA-seq reference dataset, CLIMB retained high accuracy whereas both NNLS implementations performed poorly (S9B Fig). This difference was observed specifically when reference and target data were generated using different technologies. Consistently, expression correlations between average cell-type profiles and pseudo-bulk samples were substantially higher than those observed with real bulk RNA-seq samples (S9C Fig), and pseudo-bulk and real bulk samples themselves showed weak expression correlation (S9D Fig). Together, these results show that NNLS performance is sensitive to cross-platform discrepancies between scRNA-seq–derived reference profiles and real bulk RNA-seq data, whereas CLIMB maintains stable accuracy when applied to both pseudo-bulk and real bulk RNA-seq samples.

### Evaluating potential bias towards cellular proportions contained in reference dataset

The deconvolution process of CLIMB, which employs the entire single-cell matrix as input, may introduce bias mirroring the cellular proportions in the reference matrix. To mitigate such bias, CLIMB’s model executes three sequential deconvolutions. After deconvoluting cellular proportions during its first iteration, CLIMB subsets cells in the reference matrix based on these proportions, which subsequently serve as a reference for the next deconvolution. This process is iterated twice, progressively adjusting the reference matrix to align its cellular proportions with those of the target bulk sample (S2 Fig, Methods). To demonstrate CLIMB’s model accuracy and robustness, we studied how the correlation between cellular proportions in the reference matrix and target bulk samples impacts deconvolution accuracy. We simulated eleven scenarios ranging from strong to zero-correlation contexts, using reference and pseudo-bulk data derived from AML cells in the Van Galen et al. dataset (S3A Fig). We examined accuracy and resilience to induced bias, which we inferred from the variability in accuracy across our range of bias contexts. By comparing *CLIMB-corrected* (the default cell subtype proportions provided by CLIMB) with the first iteration of CLIMB algorithm (’CLIMB-init’) to a panel of seven deconvolution methods, we observed that *CLIMB-corrected* was the second most accurate method, and the third less biased method (S3B-S3D Fig). Interestingly, *CLIMB-corrected* demonstrated enhanced accuracy and resilience to bias over CLIMB-init in most cross-dataset pseudo-bulk analysis, particularly in cases with significant cellular proportion imbalances between reference and target bulk (S3E-S3F Fig). Overall, we demonstrate that CLIMB iterative deconvolution exhibits high accuracy while maintaining low bias.

### Benchmarking Bulk Deconvolution Methods using scRNA-seq Derived Pseudo-bulk Samples in a Cross-Dataset Context

To extend our *in-vitro* findings, we conducted cross-dataset deconvolution by integrating five pairs of scRNA-seq datasets in five cancer types, totaling ten cross-dataset analyses [28,37–44]. These cross-dataset analyses first involved transferring cell-subtype labels between datasets. Then, one dataset served as the reference single-cell dataset while the other was used to generate pseudo-bulk samples (Fig 3A). Importantly, the reference dataset and the pseudo-bulk datasets were constructed using only non-batch-corrected raw counts to preserve a realistic batch effect across datasets. The comprehensive results of this large-scale analysis demonstrated that CLIMB significantly outperformed other deconvolution methods (Fig 3B), based on an aggregated ranking across four evaluation metrics (PCC, SCC, RMSE, and R2). In the context of Acute Myeloid Leukemia (AML), CLIMB outperformed other methods (Fig 3C) in (i) ‘Van Galen> Naldini’ deconvolution, with CLIMB achieving the best PCC, SCC, and R2 (Figs 3D, 3F, S4E), (ii) ‘Naldini> Van Galen’ deconvolution, where CLIMB achieved the highest PCC, RMSE, and R2 (Figs 3E, 3F, S4G), and (iii) ‘200 simulated pseudo-bulks’, a dataset of 200 simulated bulk samples generated from the Van Galen et al. dataset, where CLIMB demonstrated the top scores for all four metrics (S4F Fig). Interestingly, CLIMB demonstrated superior performance in predicting the proportion of leukemic stem cells (‘HSC-like’ in Van Galen dataset) in both analysis with available HSC-like labels (Fig 3G). Moreover, CLIMB ranked first for CRC cancer analysis (S4H-S4J Fig). CiberSortX and CLIMB emerged as the best methods in Melanoma analysis (‘Tirosh> Jerby’ and ‘Jerby> Tirosh’, S4K-S4M Fig). In the Glioblastoma context, CLIMB was the top-performing method both in the ‘10x> SmartSeq2’ and the ‘SmartSeq2> 10x’ analysis (S4N-S4P Fig). In breast cancer context, MuSiC performed best, based on ‘Gray> Wu’ and ‘Wu> Gray’ analyses (S4Q-S4S Fig). We also analyzed a nasal swab dataset of healthy and COVID-19 samples [45], using 29 patients as an scRNA-seq reference and the remainder to generate pseudo-bulk samples. In this disease context, CLIMB showed the best deconvolution performance (S9E Fig). Overall, these findings highlight CLIMB’s top-ranking accuracy in cross-dataset pseudo-bulk analyses across diverse disease and cancer contexts.

**Fig 3:**
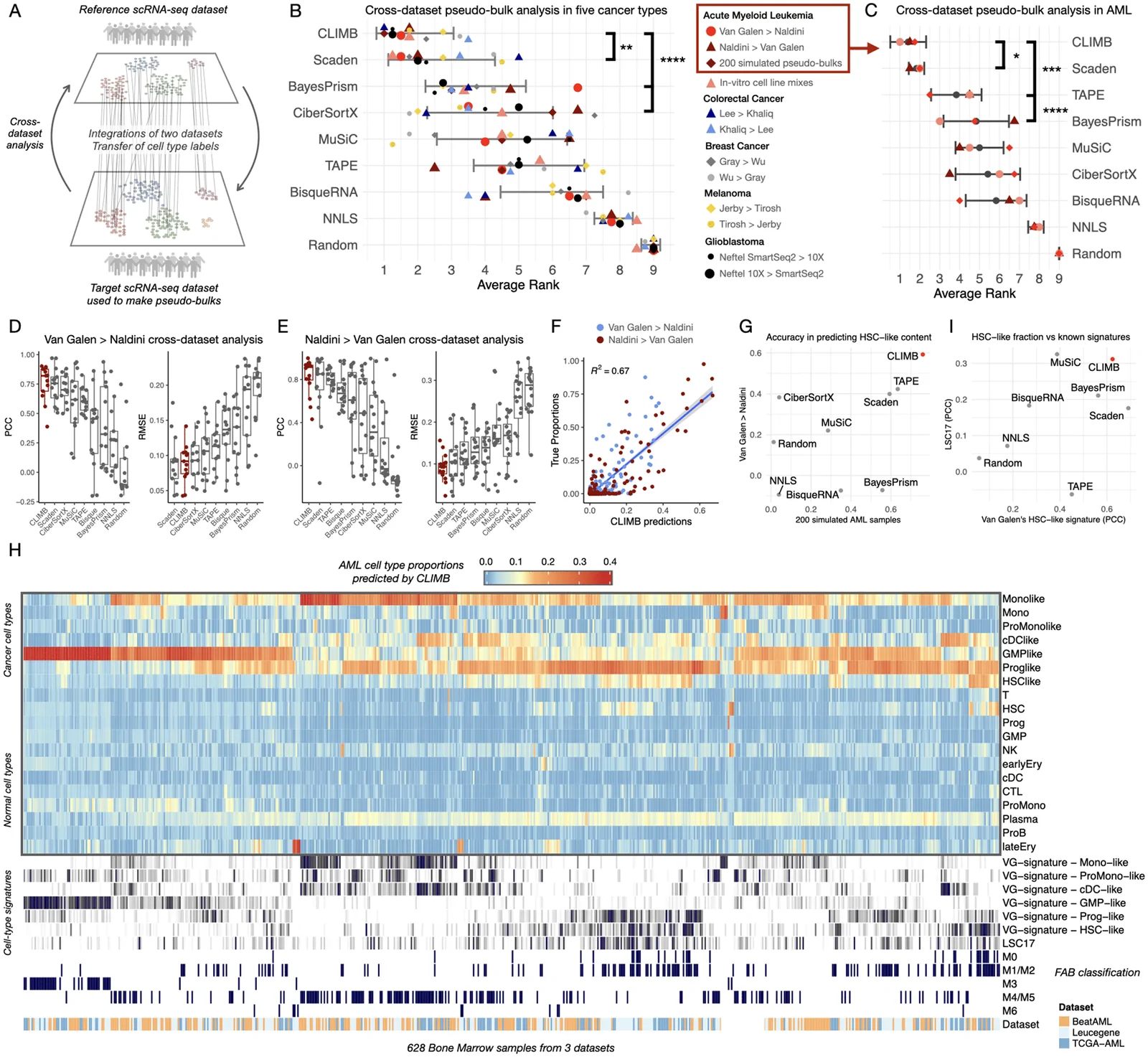
Cell Subtype Composition Deconvolution in Acute Myeloid Leukemia. **(A)** Experimental design outline for cross-dataset deconvolution analysis. **(B)** Average rank across four metrics (PCC, SCC, R2, and RMSE) for 10 cross-dataset pseudo-bulk deconvolutions, including *in-vitro* mixes and 200 simulated AML samples, totaling 12 analyses. **(C)** Subset of **(B)**, focusing on the four AML context analyses. **(D)** Pseudo-bulk analysis using Van Galen dataset as a reference for Naldini’s pseudo-bulks. Boxplots depict PCC/RMSE values for each pseudo-bulk sample (13 samples, 19 cell subtypes’ predictions). **(E)** Similar to **(D)**, but in the Naldini > Van Galen context, with 16 data points for the 16 samples in the Van Galen dataset. **(F)** Scatter plot comparing true proportions with deconvoluted proportions using CLIMB (left) and Scaden (right) for both Van Galen > Naldini and Naldini > Van Galen analyses. **(G)** Predictive accuracy of HSC-like cell subtype proportions for Van Galen > Naldini analysis, comparing 13 predicted proportions with true proportions. **(H)** Inferred cell-subtype proportions using CLIMB for bone marrow in BeatAML, Leucegene, and TCGA-AML cohorts (N = 628), along with scores of seven signatures (LSC17 and six Van Galen signatures) and FAB labels. **(I)** HSC-like fraction vs known signatures.

### Bulk deconvolution of cell subtype abundance across three aml patient cohorts

We performed deconvolution of cell subtype proportions using bulk RNA-seq samples from three AML cohorts: beatAML [12], Leucegene [46], and TCGA-AML [47], using as a reference only cells in pre-treatment conditions from Van Galen dataset [28]. Employing CLIMB, we delineated a landscape of cell subtype proportions in AML (Fig 3H). To assess deconvolution accuracy in patient samples, we juxtaposed CLIMB-derived proportions with FAB classification [48], a scheme categorizing patients according to blast (cancer cell) differentiation patterns. Specifically, blasts from M0/M1 patients, characterized by minimal differentiation, were expected to correlate with higher proportions of HSC-like and/or Progenitor-like cancer cells, a correspondence verified by CLIMB-derived proportions (Figs 3H, S5A). Similarly, M3 samples (PML-RARA classified patients) were anticipated to exhibit enrichment for GMP-like cells, in line with our deconvoluted GMP-like proportions (S5A Fig). Moreover, we observed monocyte-like cancer cells to be enriched in M4/M5 patients, along with erythrocyte enrichment in M7 patients, both of which were expected (S5A Fig). To evaluate CLIMB’s accuracy in predicting leukemic stem cells, we correlated deconvoluted HSC-like proportions with the LSC17 signature [35]. As expected, the highest correlations were observed with Prog-like and HSC-like cell subtypes (S5B Fig). Interestingly, proportions of HSC-like cells predicted by CLIMB showed a noticeable alignment with both the HSC-like signature established by Van Galen et al. [28] (S5C Fig) and the LSC17 signature, when compared to other deconvolution methods (Fig 3I). To evaluate the consistency of results across different deconvolution algorithms, we assessed cross-method correlations across all three AML cohorts. We observed that CLIMB, Scaden, and TAPE formed a distinct cluster (S5D Fig). In summation, CLIMB provides a robust depiction of the cellular landscape of AML patients across three cohorts, laying foundations for deconvoluting cell subtype drug sensitivity.

### Benchmarking bulk expression deconvolution into cell subtype expression and simulated over-expression

CLIMB enables the deconvolution of bulk-level RNA expression into cell subtype-specific expression. This can be achieved through either an ‘overall’ approach, which accounts for expression averaged across all bulks used as input, or a ‘high-resolution’ mode that disentangles cell subtype expression within each bulk sample. In our *in-vitro* experiment with leukemic cell lines, CLIMB’s ‘overall’ deconvoluted expression achieved high accuracy when compared to bulk RNA-seq expression obtained from pure cell lines (PCC of 0.94, S6A-S6C Fig). BayesPrism displayed similar patterns and accuracy to CLIMB, achieving a PCC of 0.92 (S6C Fig). Subsequently, we deconvoluted AML cell-subtype expression based on the ‘Van Galen> Naldini’ cross-dataset framework. Notably, the ‘overall’ mode of CLIMB demonstrated compelling deconvoluted expression patterns for cancer cell markers (PCC of 0.75, S6D-S6F Fig), surpassing the performance of BayesPrism (0.59) and CiberSortX in ‘group mode’. We then subjected CLIMB’s deconvoluted expression to a ‘high-resolution’ test, simulating the over-expression of 20 genes in the HSC-like subtype. This induction was applied to 7 out of 13 pseudo-bulk samples, with varying fold changes. The simulated expression was then compared to true expression values, showing CLIMB’s deconvoluted expression high accuracy at fold change 10 and higher (S6G Fig). To assess the precision in detecting differentially expressed genes (DEGs), we conducted a DE analysis between wild-type (WT) and over-expression (OE) conditions. We employed the AUC-ROC metric to evaluate DEG detection accuracy (S6J-S6K Fig). BayesPrism exhibited the highest accuracy in recovering DEGs followed by CLIMB (S6J Fig). As a positive control, we checked that true simulated expression efficiently recovered DEGs (AUC > 0.95 with fold change > 2, S6J, S6K Fig). We then computed correlations (PCC) between true and deconvoluted expression (S6L Fig), showing BayesPrism as the most accurate (median PCC of 0.76), followed by CLIMB (0.66). In summary, CLIMB demonstrates accuracy in expression deconvolution, particularly in ‘overall’ mode.

### Benchmarking drug sensitivity deconvolution with *in-vitro* cell line mixtures

In this next step, we integrate drug sensitivity data (measured as a cell viability rate) with CLIMB’s bulk deconvolution results. To achieve this, we developed CLIFF, a method that deconvolutes bulk-level cell viability into cell-subtype-specific cell viability (Figs 1, 4A). We generated drug sensitivity data from *in-vitro* mixtures comprising six combinations of four human leukemia cell lines, as previously depicted in Fig 2 (HL60, SUDHL4, K562, and THP1). These mixtures were exposed to seven drugs commonly employed in AML consolidation therapies (5-Aza-2’-deoxycytidine, cytarabine, imatinib, nilotinib, doxorubicin, rapamycin, mitomycin C). To assess the accuracy of drug sensitivity deconvolution, we determined the ground truth cell viability of the four individual cell lines when exposed individually to the same drug panel, doses, and time points. We initially evaluated the theoretical basis of CLIFF’s model by reconstructing the experimentally-assessed cell mix viability from the experimentally-determined viability of pure cell lines. This was achieved using CLIFF’s model equations (Equations 15, and 16) to correlate both metrics (S7A Fig), which showed a high Pearson’s correlation of 0.88. In the next step, we evaluated CLIFF’s deconvoluted cell viabilities against ground truth, showing a PCC of 0.80 across all 21 conditions (Fig 4B, 4C). When utilizing high-resolution cell-subtype expression as input (‘CLIFF-highres’), the accuracy of CLIFF was diminished (PCC of 0.59, Figs 4C, S7B). Subsequently, we conducted a comparison of CLIFF’s accuracy with the approach employed by Zeng et al. [49], which relies on a correlation between cell subtype proportions and bulk-level drug sensitivity; with Bottomly et al.’s approach [50], wherein the PC1 of cell subtype signatures is correlated with bulk drug sensitivity; and a linear regression using CLIMB proportions as input and bulk-level cell viability as ouput (LinReg). When assessing drugs and timepoints individually, CLIFF exhibited the highest correlation (median PCC of 0.82, Fig 4D), followed by Bottomly (0.80), ‘CLIFF-highres’ (0.68), Zeng (0.68), and LinReg (0.51). Interestingly, CLIFF is the only method that predicts cell viability rather than providing a drug sensitivity score based on correlation metrics, which results in a significantly lower RMSE compared to all other methods, both in ‘overall’ and ‘high-resolution’ modes (Fig 4E). Thus, within a framework comprising six cell mixes encompassing four cell lines, CLIFF exhibited precise deconvolution of drug sensitivity and proved effective to integrate cell subtype proportions estimated via CLIMB.

**Fig 4:**
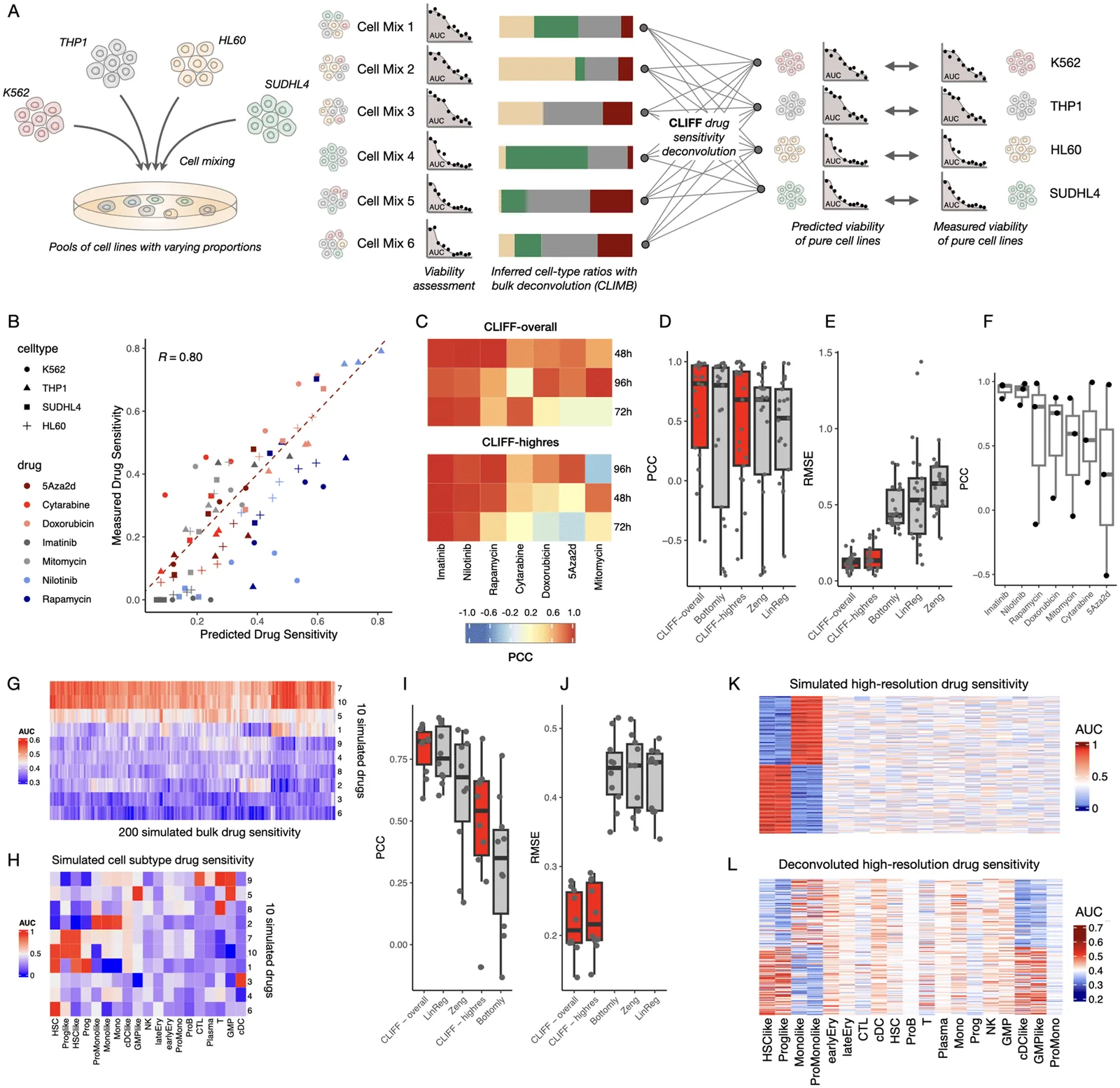
Deconvolving Cell Line Drug Sensitivity: *In-Vitro* and Simulated AML Context. **(A)** Experimental setup: Known cell line mixtures are deconvoluted to estimate cell abundances, which are integrated with bulk drug sensitivity for predicting individual cell line drug responses. **(B)** Predicted vs. actual cell line drug sensitivity for multiple timepoints, cell lines, and drugs. **(C)** Heatmap displays predictive accuracy as PCCs for each drug and timepoint. **(D)** Method comparison of deconvolution accuracy using PCCs. **(E)** Method comparison of deconvolution accuracy using RMSE values. **(F)** CLIFF’s predictive accuracy as PCC, grouped by drugs. **(G)** Drug sensitivity simulation on 200 pseudo-bulk samples derived from cell subtype sensitivities **(H)**. **(H)** Simulated cell subtype drug sensitivity pattern for 10 drugs, used for generating bulk drug sensitivity. **(I)** Predictive accuracy as PCC for CLIFF and other methods, deconvoluting cell subtype sensitivity (**G**) using bulk drug sensitivity **(H)**. **(J)** Same as **(I)**, but using RMSE metric. **(K)** Simulated high-resolution drug sensitivity across pseudo-bulk samples and cell subtypes. **(L)** Deconvoluted high-resolution drug sensitivity based on bulk samples from **(K)**.

### Simulated *Ex-Vivo* drug sensitivity assay for benchmarking deconvolution of drug sensitivity

We then simulated bone marrow samples from AML patients to generate a benchmarking dataset similar to the beatAML cohort; as the latter lacks ground truth data for testing deconvolution. Using the Van Galen single-cell dataset, we generated 200 pseudo-bulk RNA-seq samples along with their corresponding bulk cell viability data for ten simulated drugs (Fig 4G). To do this, we simulated cell viability for 19 cell subtypes across ten drugs (Fig 4H), which serves as our ground truth for evaluating CLIFF’s deconvolution accuracy. We proceeded to assess the accuracy of drug sensitivity deconvolution using the same five approaches mentioned earlier (Fig 4D–4E). In this context, CLIFF-overall demonstrated the highest accuracy, with a median PCC of 0.82 (Fig 4I), followed by LinReg (0.75), Zeng (0.68), CLIFF-highres (0.54), and Bottomly (0.35). As observed in the *in-vitro* experiment, CLIFF’s advantage, both in overall and high-resolution modes, lies in generating realistic cell viability values, which translates into significantly lower RMSE values compared to every other methods (Fig 4J). Investigating the effect of the number of input genes on CLIFF’s performance, we found that CLIFF-overall maintained consistent accuracy regardless of the gene subset (S7D Fig), while CLIFF-highres excelled particularly with higher gene counts (>500). Additionally, introducing L2 regularization improved CLIFF-highres predictions, although it had no significant impact on CLIFF-overall predictions (S7D Fig). The impact of input size on CLIFF’s accuracy was examined by varying the number of bulk samples (S7E Fig). Notably, approximately 25 cell mixes proved sufficient for CLIFF to achieve a PCC > 0.6 when deconvoluting 19 cell subtypes, and about 75 input mixtures were required to attain a PCC > 0.7. By simulating patient-specific mutations (S7F Fig) that were associated with heightened drug sensitivity in HSC-like/Prog-like cell subtypes and lowered sensitivity in Mono-like/ProMono-like cell subtypes, we examined how well CLIFF-highres could discern patient-level variability (Fig 4K, 4L). Our observations showed that CLIFF-highres detected shifts in drug sensitivity between the two sample groups in the correct cell subtypes (HSC-like, Prog-like, Mono-like, and ProMono-like). However, it also introduced false positive predictions in cDC-like and GMP-like subtypes. Benchmarking CLIFF on *in-vitro* and simulated data shows the ‘overall’ mode best predicts average cell-subtype drug sensitivity, so we restrict subsequent analyses to this mode.

### Robustness, Identifiability, and Scalability of CLIMB and CLIFF methods

We assembled a compact stress test of CLIMB and CLIFF’s ground-truth accuracy, identifiability, and computational scaling, summarized in seven panels (S10A-S10G Fig). Across 11 ground-truth datasets (in-vitro mixtures and 10 cross-dataset pairs), CLIMB’s empirical-Bayes correction improved accuracy in 9/11 cases (mean RMSE 0.095 to 0.076; S10A Fig), and this benefit extended beyond the hematopoietic tissue in which CLIMB was developed: on a non-hematopoietic dataset (COVID-19 nasal-swab), single-cell resolution alone (props.init vs. NNLS) improved with reference coverage (RMSE 0.109 to 0.096 from 5 to 500 cells/type), while NNLS remained flat (0.123-0.131; S10B Fig). Turning to CLIFF’s downstream sensitivity model, identifiability held through cross-sample cell-type proportion correlation ρ=0.8 (RMSE 0.15-0.17) before degrading sharply at ρ=0.99 (RMSE 0.26), consistent with the multicollinearity expected as the proportion matrix loses rank (S10C Fig). This near-collinear regime also made optimization less robust: 25 restarts from randomized starting coefficients converged to distinctly worse optima in a minority of cases at ρ=0.5-0.9 but a majority at ρ=0.99, though across a sweep of possible starting points, CLIFF’s default choice – starting all coefficients at zero – remained among the best-performing initializations tested (S10D-S10E Fig). On the computational side, runtime scaled approximately linearly with sample count, gene count, reference cell-type count, and reference size; at the largest scale tested (100 samples and 15,000 reference cells together), the combined climb()/cliff() pipeline completed in just over two minutes (128s), which remains practical for the dataset sizes considered here (S10F Fig). Finally, injecting calibrated proportion error matched to each method’s real accuracy showed CLIFF’s sensitivity RMSE rising monotonically from 0.158 (true proportions) to 0.185/0.194/0.231 (CLIMB-corrected/CLIMB-init/NNLS), with a non-zero floor even at perfect proportions reflecting measurement noise (S10G Fig) – underscoring that CLIFF’s accuracy is fundamentally bounded by the quality of the proportions it receives.

### Deconvoluting bulk-level drug sensitivity to cell subtype sensitivity using beatAML cohort

Utilizing cell subtype proportions and expression data estimated by CLIMB from the beatAML cohort, we integrated this information with bulk *ex-vivo* drug sensitivity data using CLIFF, which revealed a heterogeneous landscape of drug sensitivity patterns among distinct cell subtypes (Fig 5A). We assessed the findings based on 14 studies that documented cell-subtype level drug sensitivity and featured 22 pairs of drug-to-cell subtype interactions (S1 Table). CLIFF’s predictions aligned with existing literature for venetoclax [16,17], erlotinib [20], A-674563 [21], AZD1480 [22,23], and crenolanib [24], indicating elevated sensitivity in HSC-like and Prog-like cancer cells. Interestingly, midostaurin [25] and selinexor [26] were predicted to target HSC-like cell subtypes, but not progenitor cancer cells (Prog-like). We also documented drug sources that targeted differentiated blasts. Doramapimod, predicted by CLIFF to target monocyte-like cells, aligns with previous evidence of heightened activity in M4/M5 patients showing expression of CD14 monocyte marker [15]. Similar agreement was observed for trametinib [16] and selumetinib [13], known to target differentiated blasts, and projected by CLIFF to exhibit strong affinity for monocyte-like cancer cells. Bromodomain and Extraterminal Domain Inhibitors (BETi) were also shown by Romine et al. [14] to target differentiated blasts, which coroborates CLIFF’s predictions on BET-inhibitor JQ1, predicted to preferentially target monocyte-like cancer cells. Ruxolitinib [16], however, did not display a clear pattern, as it was predicted to target pro-monocyte-like but not monocyte-like cancer cells. CLIFF’s predictions also indicate a notable resistance of monocyte-like cells to venetoclax, validated by four distinct studies [15–19]. Among the 22 documented instances of cell subtype-specific drug sensitivity, CLIFF demonstrated 16/22 cases with clear concordance (72.7%), 3/22 with partial alignment, and 3/22 in direct contradiction (13.6%). Furthermore, we evaluated the concordance between CLIFF’s predictions and results reported by Bottomly et al. using the same dataset [50], revealing an overall correlation of 0.44 for all drugs (S8A Fig), with stronger correlations observed for established drug-to-cell subtype associations (S8B Fig).

**Fig 5:**
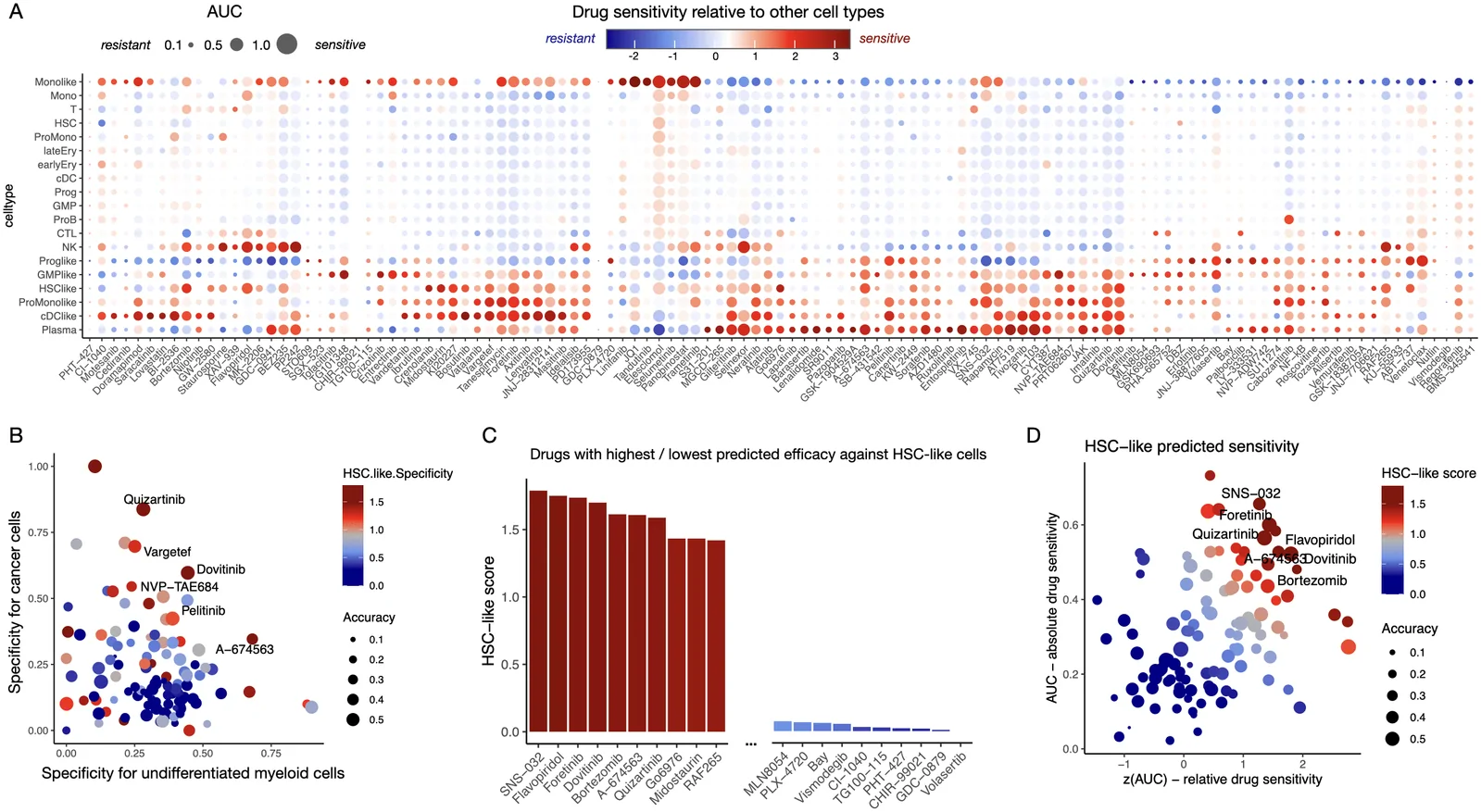
Deconvolving Cell Subtype-Specific Drug Sensitivity in AML *ex-vivo* Data. (**A)** Deconvoluted drug sensitivity across cell subtypes in the beatAML cohort dataset, with dot size indicating AUC of sensitivity and color representing standardized AUC, indicating relative drug sensitivity among cell subtypes for a specific drug. **(B)** Differentiated (Mono/ProMono/cDC) vs. undifferentiated (HSC/Prog/GMP) myeloid cells’ sensitivity (grouped) on the x-axis and normal vs. cancer myeloid cells’ sensitivity on the y-axis. **(C)** HSC-like sensitivity score computed from AUC and standardized AUC. **(D)** Drug sensitivity of HSC-like cells shown on a scatter plot with AUC on the x-axis, standardized AUC on the y-axis, and color indicating the combined score.

Subsequently, we focused on evaluating drug sensitivity in HSC-like cells, ranking drugs according to their potential effectiveness against this particular cell subtype (Fig 5B–5D). Among the top ten drugs, only A-674563 and midostaurin had available validation from the literature (S1 Table). The eight undocumented drugs predicted to be the most effective against HSC-like cells were, in order, SNS-032, flavopiridol, foretinib, dovitinib, bortezomib, quizartinib, Go6976, and RAF265. In summary, the combined use of CLIMB and CLIFF successfully unveiled a comprehensive landscape of drug sensitivity at the cell subtype level in AML, along with the ability to predict drug sensitivity in the clinically relevant HSC-like cell subtype.

### Single-cell resolution prediction identifies resistant cancer cell subpopulations

We further developed CLIFF-SC, which computes drug sensitivity prediction at single-cell resolution (Fig 6A, Methods), leveraging coefficients derived by CLIMB for each individual cell. We focused on three drugs predicted to target the HSC-like compartment: venetoclax, A-674563, and foretinib, and one drug with an opposite pattern, nilotinib, which targets monocyte-like cancer cells (Fig 5A). We visualized single-cell survival probability on 2D projections (UMAP) using all cancer cells from Van Galen dataset [28], which showed considerable heterogeneity in distributions across the four drugs and the six cancer cell subtypes (Fig 6B–6E). We observed high concordance between average cell subtype sensitivity predicted by CLIFF-SC and the one predicted by CLIFF-overall (Fig 6F). Interestingly, we observed a long upper tail of seemingly resistant HSC-like cells for venetoclax compound (Fig 6B, right panel, circled in red). We thus decided to study in more detail this population of resistant HSC-like. We performed differential expression analysis between resistant and sensitive HSC-like populations, which were clustered using single-cell venetoclax sensitivity predictions (25% most sensitive versus 25% most resistant, Fig 6G). We could show a downregulation of NPM1 expression and upregulation of ANXA1 and TMSB4XP8 in the 25% with highest predicted resistance to venetoclax. These data suggest that expression of these genes could be predictive of venetoclax sensitivity at single-cell level.

**Fig 6:**
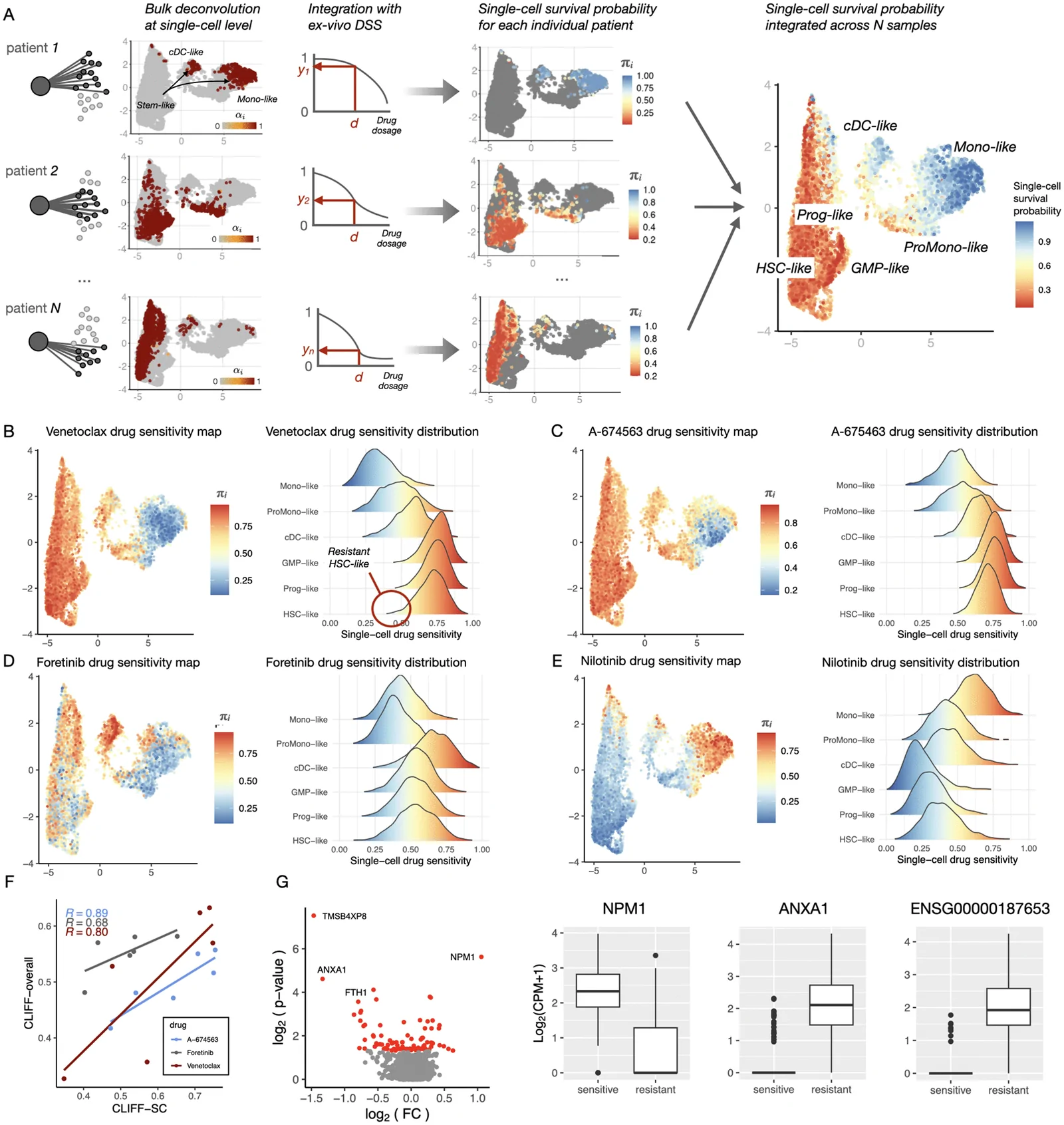
Single-Cell Resolution Drug Sensitivity Prediction. **(A)** Framework overview: CLIMB deconvolution generates coefficients for each single cell (left), integrated with *ex-vivo* drug screening data (DSS) for single-cell survival probabilities (right). **(B)** Single-cell survival probability for venetoclax, based on UMAP projections of cancer cells from Van Galen et al. [28], highlighting upper tail of resistant HSC-like cells in red circle, and survival probability distributions among six cancer cell subtypes (right). **(C)** Similar view for A-674563 single-cell drug sensitivity. **(D)** Similar view for foretinib single-cell drug sensitivity. **(E)** Similar view for nilotinib single-cell drug sensitivity. **(F)** CLIFF-overall vs. CLIFF-SC concordance scatter plot with correlation coefficients. **(G)** Left, volcano plot showing gene expression comparison between venetoclax-resistant (25%) and sensitive cells (25%) with p-value versus log fold change. Right, 3 genes misregulated in venetoclax-resistant population of HSC-like cells.

## Discussion

Recent studies have highlighted associations between cellular composition and drug response in AML, demonstrating that differentiation state influences treatment sensitivity [50] and that specific cell subtypes correlate with therapeutic response or resistance [49,51]. Notably, these analyses relied on correlations between bulk-level drug sensitivity measurements and inferred cell subtype proportions, without explicitly deconvolving drug sensitivity at the cellular level. This limitation underscores the need for computational approaches capable of resolving bulk functional readouts into interpretable cell-type–specific metrics. To address this challenge, we introduce a computational framework designed to decompose bulk-level cell viability measurements into cell subtype– and single-cell–resolved drug sensitivity profiles. The framework consists of two sequential components, CLIMB and CLIFF, which together enable bulk-to-single-cell deconvolution using paired bulk RNA-seq and bulk drug sensitivity data (Figs 1, S1, S2).

The beatAML consortium achieved the most extensive *ex-vivo* drug sensitivity screening (DSS) to date across all cancer types [12], paving the way for deciphering cell subtype-specific drug sensitivity. Our study proposes a method to address this challenge, potentially replacing resource-intensive investigations that would be required for assessing cell population-level drug sensitivity in large patient cohorts. We present a pioneering approach to deconvolute bulk drug sensitivity data into its constituent cell subtypes, employing a method capable of retrieving realistic AUC metrics for cell subtype-level drug sensitivity. Through extensive evaluation in cell line mixes and acute myeloid leukemia (AML), we predict drug sensitivity for 19 cell subtypes, 121 anti-cancer drugs, and 286 patients, culminating in a comprehensive dataset comprising over 344,318 data points derived from the beatAML dataset.

Bulk deconvolution is a cost-effective method to uncover cell composition in patient cohorts. To this end, we introduce CLIMB, a novel algorithm that outperforms current standards in various contexts, achieving accuracy comparable to deep learning-based methods with a seemingly simple linear model. CLIMB’s advantage lies in its use of the complete single-cell expression matrix, unlike other methods that uses average cell subtype expression across bulk samples. This is crucial, as cancer cell expression variability among samples is expected. Based on or cross-dataset pseudo-bulk study, CLIMB seems to effectively address batch effects. Notably, CLIMB excels in estimating proportions of HSC-like cells, a subtype linked to poor AML prognosis and relapse, enhancing predictions of their drug sensitivity. We anticipate CLIMB’s utility across diverse biological scenarios, particularly in deciphering cancer sample cellularity.

While a simple non-negative least square (NNLS) algorithm achieves high accuracy in deconvoluting cell subtype fractions from pseudo-bulk samples where reference and target data are generated using the same scRNA-seq technology, its marked failure on real bulk RNA-seq reveals a strong sensitivity to cross-platform discrepancies. These technology-induced biases likely arise from multiple sources, including pervasive gene dropouts and zero inflation in scRNA-seq, differences in transcript capture efficiency and dynamic range, platform-specific library preparation and normalization effects, or gene-level biases. In addition, NNLS relies on averaged cell-type expression profiles, an assumption that may fail in the presence of substantial within–cell-type heterogeneity. By contrast, CLIMB mitigates both reference composition bias and technology mismatch by leveraging the full single-cell expression matrix and iteratively adjusting reference cell-type proportions to better align with the target bulk sample. These findings suggest that the combination of technology-induced distortion and information loss due to cell-type averaging underlies NNLS underperformance, whereas CLIMB’s iterative, cell-resolved framework provides a more robust approach for deconvolving real bulk RNA-seq data.

By considering latent variables and employing an Expectation-Maximization algorithm, CLIFF deconvolves bulk-level cell viability into its sub-population-level components (Method). We propose that the primary strength of the CLIFF method, when compared to prior methodologies [49–51], lies in its capacity to generate biologically interpretable metrics of cell viability at the cellular level. This stands in contrast to correlative techniques that merely provide a score linking cell subtypes and drugs without delivering a meaningful biological measure. We demonstrated CLIFF abilities first with engineered mixes of leukemic cell lines and pseudo-bulk analysis. We explored multiple avenues for extending CLIFF’s ‘overall’ mode–-which provides an averaged prediction across all bulk samples taken as input–-with two higher resolution strategies. On one hand, CLIFF-highres, which uses cell subtype expression and mutation data as input, delivering cell viability predictions for each sample. In our benchmarking, CLIFF-highres generally showed decreased accuracy compared to CLIFF-overall, likely due to the limited number of input samples. In contrast, CLIFF-SC employs a full-sized single-cell dataset as input, substituting cell subtypes with single cells in CLIFF’s original model, thereby allowing the prediction of single-cell drug sensitivity. It proved to be a straightforward method to enhance CLIFF’s resolution, providing valuable single-cell maps of drug sensitivity. We then turned to the beatAML dataset, using CLIFF-overall model enhanced with mutation data as covariates. In this setting, we could reveal an extensive landscape of cell subtype drug sensitivity. Also, we predicted A-674563, Midostaurin, SNS-032, and foretinib, among other compounds, to be efficient at targeting HSC-like cells. We validated our findings by relying on 14 publications that documented cell-subtype level drug sensitivity through functionnal experiments in AML patient’s samples, for 22 pairs of drug to cell subtype interactions [13,15–17,20–27], which showed overall good agreement with our predictions (72.7%).

Beyond this validation, we further characterized three properties that govern CLIFF’s robustness in practice: identifiability, sensitivity of its EM algorithm to initialization, and how uncertainty in upstream proportion estimates propagates into its predictions. This relies on the sample-by-cell-subtype proportion matrix having full column rank, a condition that is violated when subtype proportions are strongly correlated across samples or when a subtype has near-constant proportion, both of which inflate the variance of the recovered coefficients. Simulations confirm CLIFF remains accurate up to correlation ρ≈0.8 (RMSE 0.15–0.17) but degrades sharply by ρ=0.99 (RMSE 0.26; S10C Fig). CLIFF’s EM optimization is nonconvex; random-restart experiments show local optima become more frequent as identifiability decreases, though the default (zero) initialization performs comparably to typical restarts (S10D-S10E Fig). Accuracy remains robust to initialization except when identifiability is poor and the initial spread of CLIFF’s beta coefficients is large. To quantify how proportion uncertainty propagates into CLIFF, we injected calibrated proportion errors – matching the accuracy of true, CLIMB-corrected, CLIMB-uncorrected, and NNLS estimates – into a well-identified simulation. Downstream sensitivity error increased monotonically (RMSE 0.158–0.231) but less than proportionally to the upstream proportion error, and remained above zero even with exact proportions, reflecting measurement noise in the drug-sensitivity data itself (S10G Fig).

Our approach has some limitations. First, since ground truth cell viability data is not available for patient samples, it is challenging to assess our deconvolution results on AML cohorts. This limitation led us to use a small compilation of literature as a comparison point. Second, any weakness in CLIMB’s model could potentially affect CLIFF’s predictive accuracy. We believe CLIFF would benefit significantly from being used in contexts where cellular proportions can be directly observed and measured rather than predicted through deconvolution. For example, using a cohort of patients with available scRNA-seq data and cell viability measures. Third, we rely on cell viability observed *ex-vivo* from bone marrow samples in 96-well cell viability plates, an environment which might bias the viability of fragile cell subtypes. Fourth, CLIMB’s iterative reweighting corrects for cell types that are present but over- or under-represented in the reference (S3 Fig); it cannot recover a cell type that is absent or severely depleted. This is a particular concern for non-hematopoietic (solid) tissues, where dissociation is known to systematically deplete specific, fragile cell types [52] and to induce stress-response transcriptional artifacts [53], unlike blood or bone marrow where cells are naturally in suspension.

By integrating diverse data modalities, our combined approach seeks to enhance cellular composition predictions and unravel drug sensitivity at the level of individual cell subpopulations. Here, we make a pioneering effort, as far as our knowledge extends, to advance drug sensitivity deconvolution to novel resolutions, encompassing personalized and single-cell-level predictions. As the integration of single-cell RNA-seq and *ex-vivo* drug sensitivity assays gains traction for diagnostic applications, the development of suitable computational methodologies becomes imperative to fully leverage the wealth of information from these joint data sources.

## Methods

### Gene and cell selection

In our approach to gene subset selection prior to bulk deconvolution, we utilized the Scran::ModelGenVar function on the expression matrix derived from single-cell RNA sequencing (scRNA-seq) data. By applying this methodology, we identified genes with estimated biological variance surpassing technical variance, as indicated by positive biological variance outcomes from ModelGenVar. This strategy eliminated the reliance on arbitrary thresholds for the determination of variable or deferentially expressed genes and was consistently employed across all analyses in this study.

Regarding cell selection, in the context of our *in-vitro* experiment, we observed cells forming five clusters, with four clusters being distinctly attributed to the four mixed cell lines. The fifth cluster lacked a clear marker expression pattern, exhibited smaller size (only few cells) compared to the other clusters, and displayed elevated mitochondrial read content. Consequently, we excluded this ambiguous cluster from our analysis, while retaining the remaining cells in four clusters that were attributed their known cell line labels according to their marker expression pattern.

Regarding the re-analysis of publicly available scRNA-seq datasets, we harnessed the cell selection information provided in the processed data tables, abstaining from further cell filtering based on specific criteria. In instances where datasets encompassed over 30,000 cells, we subsampled them to reach a count of 30,000 cells, adopting the set.seed(1) option for reproducibility. This enabled us to execute the Seurat integration tool without memory constraints. The datasets subjected to subsampling included Naldini et al., Gray et al., Wu et al., and Khaliq et al.

### Prototypical bulk deconvolution model

The goal of a bulk deconvolution model is to fit coefficients $w_{k}$ corresponding to each cell-subtype *k* through a linear model. Fitted coefficients directly relates to cell-subtype proportions. Thus, bulk deconvolution solves the following problem:


$$\begin{array}{c} {𝐱}_{n}\approx 𝐒\cdot {𝐰}_{n} \end{array}$$ (1)

with ${𝐱}_{n}$ representing a vector of gene expression of length *G*, for a given mixture *n*. The matrix **S** is a *G* by *K* matrix containing the average expression for each cell-subtype *k* and each gene *g*. To obtain proportions $w_{nk}$ that sum up to 1, the model can either impose a constraint $\sum_{k}w_{nk}=1$, or the coefficients $w_{nk}$ can be normalized after fitting. In this model, the *g*enes are our observations, and corresponds to genes shared between ${𝐱}_{n}$ and **S** or to a subset of marker genes. Note that every mixture *n* are resolved with an independent linear model, and that the average expression of cell-subtypes is usually derived from a scRNA-seq that does not contain mixture *n*. Thus, we assume that $𝐒\approx {𝐒}_{n}$. Of note, this simple model do not apply for advanced deconvolution algorithms like CiberSortX, BayesPrism, Scaden, BLADE, or TAPE.

### CLIMB: Cell-level Linear model through bulk-to-single-cell deconvolution

We developed the CLIMB method, which proposes an innovative approach to solve a bulk deconvolution problem, by applying a mapping from each bulk sample to each single-cell from a reference dataset. We thus fit the coefficients $\alpha_{i}$ for each single-cell *i* in an intial run of CLIMB, and thus we find the best linear combination of single-cell $c_{i}$ to predict bulk expression ${𝐱}_{n}$. Thus, we use the relation ${𝐱}_{n}\sim 𝐂\cdot \alpha_{n}$, shown below in more details:


$$\begin{array}{c} \left[\begin{array}{c} x_{n1}\\ \vdots \\ x_{nG} \end{array}]\propto \underset{{\text{C}}^{\left(G\times C\right)}}{\underbrace{\left[\begin{array}{ccc} c_{1}^{1} & \ldots & c_{1}^{C}\\ \vdots & \ddots & \vdots \\ c_{G}^{1} & \ldots & c_{G}^{C} \end{array}\right]}}\}\cdot [\begin{array}{c} \alpha_{n}^{1}\\ \vdots \\ \alpha_{n}^{C} \end{array}\right] \end{array}$$ (2)

where the vector ${𝐱}_{n}$ represent the bulk expression matrix for a mixture *n*, **C** is the raw counts matrix from scRNA-seq, and $\alpha_{n}$ is the vector of coefficients fitted for each single-cell *i* for a given mixture *n*. Thus, CLIMB loss function is defined by:


$$\begin{array}{c} L(\alpha_{n}|𝐂,{𝐱}_{n})=\min_{\alpha }\frac{1}{2G}{\left|{𝐱}_{n}-𝐂\cdot \alpha_{n}\right|}^{2}\;\;\text{, with}\;\;\alpha_{ni}\in [0,+\infty ] \end{array}$$ (3)

Thus, we impose a non-negativity constraint on the coefficients, and no regularization. We optimize $\alpha_{i}$ coefficients associated with each single-cell *i* with glmnet R library [54], with the options lower.limits = 0, lambda = 0, and scale = TRUE, intersect = FALSE. We indeed observed no improvements of the deconvolution with neither *l*_1_ nor *l*_2_ regu*l*arization.

We then group and normalize coefficients $\alpha_{ni}$ obtained with (3) to obtain the cell-subtype proportions $w_{nk}$ for each cell-subtype *k* and mixture *n*:


$$\begin{array}{c} w_{nk}=\frac{\sum_{i=1}^{C}\tau_{ki}\cdot \alpha_{ni}}{\sum_{i=1}^{C}\alpha_{ni}} \end{array}$$ (4)

With $\tau_{k}$ being an indicator variable of length *C*, equal to 1 whenever cell *i* is of type *k*.

### Empirical Bayes sub-sampling mitigates bias induced by reference matrix

The equations 2, 3, 4 yield biased estimates of cellular proportions towards the cellular proportions contained in the reference single-cell matrix **C**. This bias acts as a prior, impacting CLIMB’s predictions. To enhance cellular composition depiction, we’ll correct **C** by informed cell sub-sampling, creating a new matrix via an Empirical Bayes approach estimating the prior from the data.

We use the initial CLIMB-derived cellular proportions from equations 2 3, 4 to sub-sample half of the cells in reference matrix C based on our predicted biased proportions. Each sub-sample ensures a minimum of 50 cells (or 2% of total cell count) per cell subtype. In total, five sub-sampling are done, and this process is repeated three times. Ultimately, the proportions from the second iteration - and thus third deconvolution - become the final CLIMB output (stored in ‘props.corrected’). CLIMB’s iterative algorithm is summarized in S2 Fig.

Additionally, a gene selection aids CLIMB’s deconvolution. First, dataset-specific top-variable genes are selected based on cell subtype average expression per gene. Genes with significant fold change compared to the average expression and compared to the second top-expressed cell subtype, are chosen (threshold set to 95% and 75% fold changes quantiles). cell subtypes with top expression are recorded, allowing a maximum of 100 genes per cell subtype. This initial gene selection is followed by bulk sample-specific gene selection: Genes are ranked according to their maximum cell subtype expression multiplied by their expression in the target bulk sample, and the top 500 genes are selected. These gene selection are thus bulk sample-specific.

### Deconvolution of cell-subtype expression

CLIMB allows the prediction of cancer cell expression in a two step manner. Let first define our target *high-resolution* expression ${\hat{𝐒}}_{\left(ngk\right)}$, which is the cell-subtype-specific and bulk sample-specific expression for each cell-subtype *k*. We assume that for a given group of samples, the *grouped* expression ${\hat{𝐒}}_{\left(gk\right)}$ relates to the *high-resolution* expression with the following formula:


$$\begin{array}{c} s_{gk}^{\left(grouped\right)}=\frac{1}{N}\sum_{n=1}^{N}s_{ngk}^{\left(high-res\right)} \end{array}$$ (5)

First, we take advantage of the bulk to single-cell mapping to directly derive the cell-subtype expression from the bulk-to-single-cell deconvolution or *mapping*. We can simply sum up the expression of cells mapped by CLIMB to get a *high-resolution* cell-subtype-specific and bulk-specific expression $s_{ngk}^{\left(mapping\right)}$:


$$\begin{array}{c} s_{ngk}^{\left(mapping\right)}=\sum_{i=1}^{C}\alpha_{in}\cdot c_{ig}\cdot \tau_{ik} \end{array}$$ (6)

with $\alpha_{in}$ being the coefficient fitted by GLMNET on each single-cell, linking each cell *i* to each bulk *n*. $c_{ig}$ denotes the single-cell expression of cell *i* and gene *g*, and $\tau_{ik}$ is an indicator vector equal to 1 if cell *i* is of type *k*.

Although this is potentially sufficient in cases where scRNA-seq reference dataset maps all cell states present in bulk samples, it can be insufficient to detect genes deferentially-expressed (DE) at cell-subtype level. As DE genes will likely induce error on the cell-subtype expression deconvolution, we use the error itself to fit our model. We can also make the reasonable assumption that DE genes only originates in cancer cells. Thus, for a given gene *g* and a given bulk *n*, we have:


$$\begin{array}{c} y_{ng}\sim {\hat{y}}_{ng}^{\left(mapping\right)}+\epsilon_{ng}\;\;\;\to \;\;\;y_{ng}-{\hat{y}}_{ng}^{\left(mapping\right)}=\epsilon_{ng} \end{array}$$ (7)

Thus, we use $\epsilon_{ng}=y_{ng}-{\hat{y}}_{ng}^{\left(mapping\right)}$ as input for a model to predict cancer cell-subtype expression. We decompose our error $\epsilon_{ng}$ between the error originated from DE genes $\epsilon_{ng}^{\left(DE\right)}$, and an additional error term $\epsilon_{ng}^{*}$. Similar as other methods, we use the information from all bulk samples *N* to deconvolute the high-resolution cell-subtype specific expression ${\hat{s}}_{ngk}^{\left(DE\right)}$. We consider that $\epsilon_{ng}^{\left(DE\right)}$ only originates from cancer cells *C*^(*C*)^ of type *K*^(*C*)^, thus


$$\begin{array}{c} \epsilon_{ng}\sim \underset{\epsilon_{ng}^{\left(DE\right)}}{\underbrace{\sum_{k=1}^{K^{\left(C\right)}}\left(w_{nk}\cdot {\hat{s}}_{ngk}^{\left(DE\right)}\right)}}+\;\epsilon_{ng}^{*}=\sum_{i=1}^{C^{\left(C\right)}}\left(\alpha_{in}\cdot {\hat{c}}_{ig}\right)+\epsilon_{ng}^{*}=𝐀\cdot {\hat{𝐜}}_{g}+\epsilon_{ng}^{*} \end{array}$$ (8)

Where ${\hat{𝐜}}_{g}$ is the vector of coefficients we wish to predict. We used Glmnet with options lower.limits = 0, lambda = 0, and scale = TRUE, fitting the following loss function:

Our final prediction of cell-subtype expression for a cancer cell-subtype *k*, a given bulk *n*, and a given gene *g* is:


$$\begin{array}{c} {\hat{s}}_{ngk}^{\left(high-res\right)}=\underset{\text{from scRNA-seq}}{\underbrace{{\hat{s}}_{ngk}^{\left(mapping\right)}}}+\underset{\text{learned}}{\underbrace{{\hat{s}}_{ngk}^{\left(DE\right)}}}=\sum_{i=1}^{C}\alpha_{in}\cdot (c_{ig}+{\hat{c}}_{ig})\cdot \tau_{ik} \end{array}$$ (9)

Following the assumptions made above, we set ${\hat{c}}_{ig}$ to zero for normal cells, which imply that ${\hat{s}}_{ngk}^{\left(high-res\right)}={\hat{s}}_{ngk}^{\left(mapping\right)}$ for normal cell subtypes.

### CLIFF - Cell fate inference for drug sensitivity deconvolution

Intuitively, CLIFF treats each observed bulk drug-response measurement as a blend of hidden, cell-subtype-specific responses, weighted by how much of each cell subtype is present in the sample (Fig 1B). Because we never observe which individual cells survived a given drug exposure, we treat survival as a latent variable and use an Expectation-Maximization algorithm to resolve it: the E-step asks, given the current estimate of each cell subtype’s drug sensitivity, how much of the observed bulk survival should be attributed to each subtype; the M-step then uses that attribution to re-estimate each subtype’s sensitivity, and the two alternate until the estimates stabilize. The sigmoid link (Eq. 16) simply ensures that whatever combination of expression and mutation covariates the model considers, the resulting survival probability stays within [0,1].

Here we propose to infer the drug sensitivity of individual cell subtypes using only drug sensitivity data observed on mixed cell populations, considering *K* different cell populations in *N* mixed samples, with proportions than can be defined as ${𝐰}_{n}=[w_{n1},...,w_{nK}]$, with $w_{nk}\in [0,1]$ and $\sum_{k=1}^{K}w_{nk}=1$. Drug sensitivity observed in the bulk samples we wish to demix are represented by a vector **y**^(*N*)^, with $y_{n}\in [0,1]$ being the empirical surviving rate of cells, and for sample *n*. Therefore the target matrix we wish to predict is ${\hat{𝐘}}^{N\times D}$, and the predicted values of drug sensitivity is assumed to depend on the underlying drug sensitivity of cell subtypes $\pi_{nk}\in [0,1]$ for patient *n*, and cell-subtype *k*, represented as a surviving rate of cells.

To model drug sensitivity, we assume that single-cell survival is represented as a binary output $\delta_{ni}\in \{0,1\}$ that follows a Bernoulli distribution. Thus, every single-cell *i* in patient *n* exposed to a drug can either survive ($\delta_{ni}=1$) or die ($\delta_{ni}=0$). Thus a cell *i* has a probability $P(\delta_{ni}=1)$ to survive and $1-P(\delta_{ni}=1)$ to die. Let’s say we are interested to know the survival probability $P(\delta_{ni}=1)$, given a drug dose. We could resolve the log-likelihood of the Bernoulli distribution for all the cells of a single sample *n*:


$$\begin{array}{c} {ℒ}_{n}=\sum_{i=1}^{C_{n}}\left[\delta_{ni}\cdot \log P(\delta_{ni}=1)+(1-\delta_{ni})\cdot \log(1-P(\delta_{ni}=1))\right] \end{array}$$ (10)

It turns out that in our problem, $\delta_{ni}$ cannot be directly observed. We are indeed only able to observe the overall ratio of cells that survives among all cells $C_{n}$ for a given patient *n*, which we define as $y_{n}$. It relates to the values $\delta_{ni}$ with:


$$\begin{array}{c} y_{n}=\frac{1}{C_{n}}\sum_{i=1}^{C_{n}}\delta_{ni} \end{array}$$ (11)

Thus, by multiplying (10) by $1/C_{n}$, if we assume that every cell *i* share the same average probability $P(\delta_{nid}=1)$, we can re-write (10) and obtain the following log-likelihood:


$$\begin{array}{c} y_{n}\cdot \log P(\delta_{ni}=1)+(1-y_{n})\cdot \log(1-P(\delta_{ni}=1)) \end{array}$$ (12)

As stated above, the core of the problem is to assume different survival probability for our single-cells. To slightly simplify this, we can consider a cell-subtype-specific survival probability. Thus, we consider the survival probability observed in our mixed population of cells $P(\delta_{nid}=1)$, as a mixture of *K* probabilities $P(\delta_{nid}^{\left(k\right)}=1)$, one for each cell subtype. Let introduce $\pi_{kn}$, a variable describing the survival probability of cell-subtype *k*:


$$\begin{array}{c} \pi_{kd}=P(\delta_{ni}^{\left(k\right)}=1) \end{array}$$ (13)

We also define $\nu_{nd}$, which corresponds to the expected number of cells surviving in patient *n* with drug dose *d*, and assume it follows a Binomial distribution, with $y_{nd}$ being the ratio of surviving cells among cells in $C_{n}$. We further define ${\hat{y}}_{nd}$ as an average of cell-subtype survival probabilities $\pi_{kdn}$ constituting the bulk, weighted with cell-subtype proportions $w_{nk}$:


$$\begin{array}{c} \nu_{n}=C_{n}\cdot \underset{{\hat{y}}_{n}}{\underbrace{\sum_{k=1}^{K}w_{nk}\cdot \pi_{kn}}} \end{array}$$ (14)

with $w_{nk}$ the cell-subtype proportions. ${\hat{y}}_{n}$ is the predicted cell surviving rate in the sample *n*, and thus the target we wish to predict through a cross-validation procedure. We can say that if we let:


$$\begin{array}{c} {\hat{y}}_{n}=\sum_{k}w_{nk}\cdot \pi_{kn}\;\;\text{we have that}\;\;{\hat{y}}_{n}\sim Ber({\hat{y}}_{n}). \end{array}$$ (15)

Additionally, we impose the cell-subtype-specific survival probabilities $\pi_{kdn}$ to be non-linearly related with cell-subtype gene expression ${𝐬}_{kn}$ (a vector of length G) through a sigmoid function:


$$\begin{array}{c} \pi_{kn}=\sigma (\beta^{T}{𝐬}_{kn}+\Theta_{mn}) \end{array}$$ (16)

Note that the sigmoid in (16) also optionaly depends on $\Theta_{mn}$, which is an intersect associated with each mutation *m* in each patient *n*, attributed to the value 1 in each cancer cell subtype and to 0 in normal cell subtypes. To clarify the notation, we set that $\tilde{\beta }=\{\beta ,\theta \}$, with θ being a vector containing coefficients associated with each mutation *m*.

When running CLIFF in *overall* mode, there are two assumptions that we use to simplify the problem. First, we assume that every sample *n* share the same cell-subtype expression $s_{gk}$, for a given gene *g* and cell-subtype *k*. Thus, $s_{gk}\approx {𝔼}_{N}[s_{gkn}]$. Second, we assume that cells of the same cell-subtypes across the *N* samples share the same sensitivities $\pi_{k}$. We thus assume that $\pi_{k}$ correspond to the expected drug sensitivity over *N* samples, and therefore that $\pi_{k}\approx {𝔼}_{N}[\pi_{kn}]$.

Thus, the overall log-likelihood for our problem is:


$$\begin{array}{c} ℒ(\tilde{\beta }\;|\;𝐘,𝐖,𝐒)=\max_{\tilde{\beta }}\sum_{n=1}^{N}\sum_{d=1}^{D}[y_{nd}\log(\sum_{k=1}^{K}w_{nk}\cdot \pi_{kn}(\tilde{\beta }))+(1-y_{nd})\log(1-\sum_{k=1}^{K}w_{nk}\cdot \pi_{kn}(\tilde{\beta }))] \end{array}$$ (17)

As this log-likelihood expression contains the log of a sum, it is difficult to optimize with respect to $\tilde{\beta }$. Therefore, we use an Expectation-Maximization to iteratively define lower bound functions that we optimize with respect to $\tilde{\beta }$, which allows solving (17).

### Expectation-Maximization algorithm to solve CLIFF

By using the concavity of the log through Jensen inequality, we can define a lower bound of (17):


$$\begin{array}{cc} {ℒ}_{B}= & \sum_{n=1}^{N}\sum_{k=1}^{K}q_{nk}^{\left(1\right)}\cdot y_{n}\cdot \log(\pi_{kn}(\tilde{\beta }))+q_{nk}^{\left(0\right)}\cdot (1-y_{n})\cdot \log(1-\pi_{kn}(\tilde{\beta }))\\ & -\sum_{n=1}^{N}\left[y_{n}\cdot KL({𝐪}_{n}^{\left(1\right)}\|{𝐰}_{n})+(1-y_{n})\cdot KL({𝐪}_{n}^{\left(0\right)}\|{𝐰}_{n})\right] \end{array}$$ (18)

In order to solve (17), we will iterate over the Expectation step or E-step where we update $q_{nk}^{\left(0\right)}$ and $q_{nk}^{\left(1\right)}$ in (18), and the Maximization step or M-step, where we maximize (18) with respect to $\tilde{\beta }$. We can therefore define our Maximization algorithm as follow:

#### E-step.

During E-step, we define our lower bound function at step *t*, ${ℒ}_{B}^{\left(t\right)}$ by defining $q_{nk}^{\left(0),(t\right)}$ and $q_{nk}^{\left(1),(t\right)}$ using $\pi_{kn}^{\left(t-1\right)}$ defined at previous step:


$$\begin{array}{cc} & q_{nk}^{\left(1),(t\right)}=\frac{w_{nk}\cdot \pi_{kn}^{\left(t-1\right)}}{\sum_{k=1}^{K}w_{nk}\cdot \pi_{kn}^{\left(t-1\right)}}\\ & q_{nk}^{\left(0),(t\right)}=\frac{w_{nk}\cdot (1-\pi_{kn}^{\left(t-1\right)})}{\sum_{k=1}^{K}w_{nk}\cdot (1-\pi_{kn}^{\left(t-1\right)})} \end{array}$$ (19)

This gives us the lower bound function of (18), tight to the original log-likelihood (17) when $q_{nk}^{\left(0),(t\right)}$ and $q_{nk}^{\left(1),(t\right)}$ satisfies (19).

#### M-step.

During M-step, we optimize (18) with respect to $\tilde{\beta }$, considering the variables **q**^(0), (*t*)^ and **q**^(0), (*t*)^ defined during E-step as constants. Also, the two last term of (18) do not depend on $\tilde{\beta }$, thus we can solve the following problem which will produce the same optimal $\tilde{\beta }$ coefficients than if solving (18):


$$\begin{array}{cc} & {ℒ}_{B}=\max_{\tilde{\beta }}\sum_{n=1}^{N}\sum_{k=1}^{K}q_{nk}^{\left(1\right)}\cdot y_{n}\cdot \log(\pi_{kn}(\tilde{\beta }))+q_{nk}^{\left(0\right)}\cdot (1-y_{n})\cdot \log(1-\pi_{kn}(\tilde{\beta })) \end{array}$$ (20)

Interestingly, (20) can be solved with standard libraries using a weighted logistic regression model, where we define weights as being $\gamma_{k}^{\left(1\right)}=\sum_{n=1}^{N}q_{nk}^{\left(1\right)}\cdot y_{n}$ and $\gamma_{k}^{\left(0\right)}=\sum_{n=1}^{N}q_{nk}^{\left(0\right)}\cdot (1-y_{n})$.

We optimize $\tilde{\beta }$ coefficients through a ten-fold cross-validation (taking 90% of the samples N in the training set, and 10% in the testing set), and using early-stopping to terminate the EM algorithm. Optimized $\tilde{\beta }$ coefficients allow to compute optimized values for $\pi_{kn}$, our estimate of cell-subtype drug sensitivity.

### CLIMB-SC - bulk deconvolution at single-cell level

The bulk deconvolution performed by CLIMB has the particularity of estimating coefficient directly on single-cells from a reference scRNA-seq dataset. Instead of transforming these coefficients into cell-subtype proportions as in Methods (3), we can compute normalized coefficients ${\tilde{\alpha }}_{ni}$ with the following formula:


$$\begin{array}{c} {\tilde{\alpha }}_{ni}=\frac{\alpha_{ni}}{\sum_{i=1}^{C}\alpha_{ni}} \end{array}$$ (21)

With $\alpha_{ni}$ being the coefficients fitted with GLMNET library. We therefore call this *single-cell bulk deconvolution*, which links every bulk RNA-seq *n* to every single-cells *i* from a scRNA-seq dataset, through what we will call *single-cell proportions* ${\tilde{\alpha }}_{ni}$. These *single-cell proportions* are thus normalized coefficients fitted by CLIMB, as shown above in (21). Thus we obtain that $\sum_{i=1}^{C}{\tilde{\alpha }}_{ni}=1$. As ${\tilde{\alpha }}_{ni}$ sum up to 1, they can be used by CLIFF as if they were cell-subtype proportions.

### CLIFF-SC for drug sensitivity inference at single-cell level

By slightly modifying CLIFF method and by linking it with the ouput of CLIMB-SC, we propose CLIFF-SC, a method providing drug sensitivity estimates at single-cell resolution. If the input dataset is composed of paired bulk RNA-seq and drug sensitivity screening data, a so-called *bulk deconvolution at single-cell level* is performed using a reference scRNA-seq dataset (cf. CLIMB-SC above).

Thus, as in (15), we model the observed ratio of cells $y_{nd}$ that survives in a mixed population of cells as an average of single-cell survival probabilities $\pi_{id}$, associated with the bulk sample *n*, weighted by the coefficients ${\tilde{\alpha }}_{ni}$:


$$\begin{array}{c} {\hat{y}}_{nd}=\sum_{i=1}^{C}{\tilde{\alpha }}_{ni}\cdot \pi_{id} \end{array}$$ (22)

With *C* being the number of cells contained in a scRNA-seq reference datasets. Thus, we model the single-cell drug sensitivity $\pi_{id}$, which is also modeled with a sigmoid function, similar to (16):


$$\begin{array}{c} \pi_{id}=\sigma (\beta^{T}{𝐜}_{i}+\theta_{d}) \end{array}$$ (23)

Where ${𝐜}_{i}$ is the *i*th row of the single-cell expression matrix **C**, thus ${𝐜}_{i}$ is a vector of length *G*, representing the gene expression of cell *i*. Similarly as in the cell-subtype-centered model, we can derive a log-likelihood function:


$$\begin{array}{c} ℒ(\tilde{\beta }\;|\;𝐘,𝐖,𝐂)=\max_{\tilde{\beta }}\sum_{n=1}^{N}\sum_{d=1}^{D}[y_{nd}\log(\sum_{i=1}^{C}{\tilde{\alpha }}_{ni}\cdot \pi_{id}(\tilde{\beta }))+(1-y_{nd})\log(1-\sum_{i=1}^{C}{\tilde{\alpha }}_{ni}\cdot \pi_{id}(\tilde{\beta }))] \end{array}$$ (24)

A lower bound can be derived from (24), which gives:


$$\begin{array}{cc} {ℒ}_{B}= & \sum_{d,n}^{D,N}\sum_{i=1}^{C}q_{ndi}^{\left(1\right)}\cdot y_{nd}\cdot \log(\pi_{id}(\tilde{\beta }))+q_{ndi}^{\left(0\right)}\cdot (1-y_{nd})\cdot \log(1-\pi_{id}(\tilde{\beta }))\\ & -\sum_{d,n}^{D,N}\left[y_{nd}\cdot KL({𝐪}_{nd}^{\left(1\right)}\|{𝐰}_{n})+(1-y_{nd})\cdot KL({𝐪}_{nd}^{\left(0\right)}\|{𝐰}_{n})\right] \end{array}$$ (25)

Then, the Expectation-Maximization algorithm can initialized with all coefficients $\tilde{\beta }$ at 0. This implies that $\pi_{id}^{\left(t=0\right)}=0.5$ for all *i*, *d*. During E-step, we compute values for $q_{ndi}^{\left(0\right)}$ and $q_{ndi}^{\left(1\right)}$ variables:


$$\begin{array}{cc} & q_{ndi}^{\left(1),(t\right)}=\frac{{\tilde{\alpha }}_{ni}\cdot \pi_{id}^{\left(t-1\right)}}{\sum_{i=1}^{C}{\tilde{\alpha }}_{ni}\cdot \pi_{di}^{\left(t-1\right)}}\\ & q_{ndi}^{\left(0),(t\right)}=\frac{{\tilde{\alpha }}_{ni}\cdot (1-\pi_{id}^{\left(t-1\right)})}{\sum_{i=1}^{C}{\tilde{\alpha }}_{ni}\cdot (1-\pi_{id}^{\left(t-1\right)})} \end{array}$$ (26)

Then, we optimize $\tilde{\beta }$ coefficients at each step (t), during the so-called M-step:


$$\begin{array}{cc} & {ℒ}_{B}=\max_{\beta ,\theta }\sum_{d,n}^{D,N}\sum_{i=1}^{C}q_{ndi}^{\left(1\right)}\cdot y_{nd}\cdot \log(\pi_{id}(\tilde{\beta }))+q_{ndi}^{\left(0\right)}\cdot (1-y_{nd})\cdot \log(1-\pi_{id}(\tilde{\beta })) \end{array}$$ (27)

And we run the EM algorithm in a similar way than shown above for CLIFF.

### Simulation of pseudo-bulk samples from scRNA-seq

We generated pseudo-bulk samples by summing up single-cell expression vectors per gene to obtain an artificially generated “pseudo”-bulk RNA-seq. As the precise amount of cells from each cell subtype added to the mix is known, we thus precisely know the cell-subtype proportions of the mixture and can benchmark deconvolution algorithms efficiently. We generated two datasets of pseudo-bulk in this work, both based on single-cells found in the Van Galen et al. dataset.

First, we used all single cells from the same sample (thus, the same patient), which we summed up to obtain a patient-specific pseudo-bulk RNA-seq sample. Thus, we define a variable $\phi_{ni}$ as a binary output variable indicating if a cell *i* belongs to patient *n*. Thus, a pseudo-bulk samples is a vector of length *G* containing the gene expression summed over all cells from patient *n*:


$$\begin{array}{c} {\tilde{x}}_{n}=\sum_{i=1}^{C}\phi_{ni}\cdot {𝐜}_{i} \end{array}$$ (28)

where ${𝐜}_{i}$ is a vector of length *G* representing the gene expression of cell *i*. We can therefore easily get the cell-subtype proportion $w_{nk}$, by simply counting the number of cells from each cell subtypes and dividing by the total number of cells in patient *n*. For this we introduce an indicator vector $\tau_{k}$, which is of length *C* and equal to 1 whenever cell *i* is of type *k*.


$$\begin{array}{c} w_{nk}=\frac{\sum_{i=1}^{C}\phi_{ni}\cdot \tau_{ik}}{\sum_{i=1}^{C}\phi_{ni}} \end{array}$$ (29)

Van Galen et al. dataset offers only a limited number of patients (16 AML patients at day 0 pre-treatment). To simulate an *in-silico* a dataset of similar size to the beatAML cohort, we generated 200 pseudo-bulk samples from the Van Galen et al. dataset allowing a random mix of cells from multiple patients. First, we generated 200 random cell-subtype proportions using a Dirichlet distribution. Thus, we assume that cell-subtype proportions ${𝐰}_{n}\sim Dir(\alpha )$, with α being a vector of length *K*, containing one value for each cell subtype *k*. We fitted α using cell-subtype proportions in Van Galen et al. dataset with the R library *feralaes/dampack*. Then, we generated cell-subtype proportions for 200 pseudo-bulks, with the R library *DirichletReg* and the α previously fitted. Cells from Van Galen et al. dataset were selected with probabilities corresponding to cell-subtype proportions. To use a realistic number of cells to generate our pseudo-bulk samples, we used a gamma distribution fitted on Van Galen et al. dataset using *fitdistr* function from R library *MASS*. In the end, we generated a matrix of pseudo-bulk expression ¯𝐗 of size (N×G) with *N* = 200, together with associated true cell-subtype proportions ¯𝐖 of size (N×K).

### Simulation of drug sensitivity screening data

To simulate drug screening data, we assumed that the drug sensitivity observed on a mix of cells corresponds to the sum of the cell-subtype drug sensitivity constituting its bulk weighted by their respective proportions. Additionally, we added Gaussian noise with a signal-to-noise ratio of 100. Thus, the simulated survival rate for pseudo-bulk *n*, in cell-subtype *k* exposed to drug dose *d* was generated with the following formula, given the proportions:


$$\begin{array}{c} ¯\pi_{kd}=\frac{1}{1+exp(\zeta_{f}\cdot d+c)} \end{array}$$ (30)

with $\zeta_{f}$ being a randomly generated number, with $\zeta_{f}\in [1,2]$ if *f* = 1 (low sensibility cell-subtypes - resisting cells) and $\zeta_{f}\in [5,6]$ if *f* = 2 (high sensibility cell-subtypes). Here we did not use the drug dose *d*, only the integer number related to drug dose *d* = {0, 1, ..., 6}, as we considered seven drug doses. We arbitrarily defined three cell subtypes with high sensitivity and the rest having low sensitivity. The constant *c* added was set to c=−8 as it provided visually convincing results. Thus $¯\pi_{kd}$ follows a sigmoid function that depends on drug dose *d* but in a way that is slightly different than in our model. Then, we generated the survival rate at the bulk level using the same formula as in our model but adding gaussian noise to it:


$$\begin{array}{c} ¯y_{nd}=\sum_{k=1}^{K}¯w_{nk}\cdot ¯\pi_{kd}+\epsilon \;\;\;\;\text{with}\;\;\;\;\epsilon \in 𝒩(0,\;\sigma^{2}) \end{array}$$ (31)

and σ was chosen to generate a signal-to-noise ratio arbitrarily chosen at 100.

### Area under the curve for summarizing drug sensitivity of multiple data points

As CLIFF produces *D* predictions for each cell subtype, and CLIFF-SC produces *D* predictions for every single cell, it is desired to summarize these *D* data points into a single value. The goal is to summarize cell-subtype or single-cell drug sensitivity. To summarize drug sensitivity from *D* different drug doses, we normalize drug doses so that the minimum dose = 0 and the maximum dose = 1. As the cell survival rate is ∈[0,1], we can compute an area under the curve (AUC) which will be ∈[0,1]. Conversely, we get an AUC of 1.0 if all cell survives and an AUC of 0.0 if all cells die, taking every dose into account. We computed the AUC using the function *computeAUC* from the R package *PharmacoGx*.

### Culture conditions of cancer cell lines

Human cancer cell lines (K562, HL60, THP1, SUDHL4) were purchased from the American Type Culture Collection (ATCC; Rockville, MD, USA) and cultured at 37°C in a humidified 5% CO2 environment in RPMI 1640 (HCC38) supplemented with 2mM L-glutamine, 2 g/L D-glucose, and 10% fetal bovine serum (FBS; Gibco ThermoFisher).

### Pharmaceutical compounds

Stock solutions for every agent (Table 1) were prepared using DMSO (Sigma-Aldrich; stored at -80°C), further diluted in H2O to the appropriate concentration, plated in Greiner 96-well flat bottom and the plates were stored at -20°C. The pharmaceutical compounds were screened at eight concentrations (Log concentration M = -9.205031395, -8.699881554, -8.130127907, -7.70415926, -7.209309101, -6.70415926, -6.125806332, -5.699837685, -5.204987526, -4.699837685) using a Log2-fold dilution series with matched DMSO concentration vehicle controls.

**Table 1:** Table of eight coumpounds used in the *in-vitro* drug sensitivity experiment.

CatalogID	Name	Supplier
A3656	5-Aza-2’-deoxycytidine	Sigma
C3350000	Cytarabine	Sigma
CDS022173	Imatinib	Sigma
CDS023093	Nilotinib	Sigma
HY-15142	Doxorubicin (hydrochloride)	MedChemExpress
O9512	Oxaliplatin	Sigma
R0395	Rapamycin	Sigma
Y0000378	Mitomycin	Sigma

### Bulk proportion for bulk RNAseq and drug sensitivity

Six bulks were created by mixing the four cell lines with the proportions listed in (Table 2). Pure and mixed cell populations were subjected to RNA extraction and drug sensitivity immediately after mixing.

**Table 2:** Table of cell line proportions added in tubes manually to generate the mixtures of cell lines.

Cell line	Mixture1	Mixture2	Mixture3	Mixture4	Mixture5	Mixture6
SUDHL4	0.33	0.07	0.00	0.61	0.188	0.20
K562	0.09	0.11	0.222	0.04	0.317	0.26
HL60	0.26	0.57	0.333	0.05	0.0198	0.12
THP1	0.32	0.25	0.444	0.30	0.475	0.42

### Cell viability assay

Cells were plated in the 96-well plates containing chemical compounds previously equilibrated at 37C, at a density of 1×104 cells per well in 100μl culture medium. Cell viability was assessed after drug treatment for 24, 48, 72 or 96 in duplicates hours by incubating the cells with PrestoBlue Cell Viability Reagent, (Thermo Fisher Scientific - Life Technologies A13261). PrestoBlue was at room temperature before adding to the cells in each well. The cells were incubated with 10μl PrestoBlue (10% of cell culture volume) for two hours at 37°C. The fluorescence was measured by the bottom with a 560nm excitation filter and a 590nm emission filter in a BioTek Neo HTS microplate. The acquisitions were performed with the following parameters: Gain: 54, Light Source Xenon Flash, Lamp Energy: High, Read Speed: Normal, Delay 0 msec, Mesurements/Data Point: 10, Read Height 5.25mm.

### RNA extraction and sequencing

RNA-seq Total RNA from cell lines was isolated with NucleoSpin RNA Plus kit (Machery-Nagel). cDNA was prepared with Maxima Reverse Transcriptase (Thermo Scientific). Sequencing libraries were performed with Illumina Truseq Stranded mRNA LT kit. Reads were mapped to the human (hg19) genome using Hisat2. Counts on genes were generated using featureCounts. Normalization for sequencing depth has been done using the counts on genes as library size using the TMM method as implemented in the limma package of Bioconductor.

### Single Cell RNAseq (MULTIome-Seq)

Single cell RNAseq was performed using 10x Genomics Chromium Single Cell Controller following the 10x Genomics Multiome (scRNA/scATAC-seq) demonstrated protocol: Chromium Next GEM Single Cell Multiome ATAC + Gene Expression User Guide (CG000338). To note, experimental parameters specific to the experiment were 3,000 for the Targeted Nuclei Recovery and 5 minutes for the lysis time.

### Single-cell RNA-seq analysis

Cellranger-arc was used to obtain counts on genes using default parameters. Counts were obtained using cellranger using hg19. Only uniquely mapped reads on genes that were expressed in at least 1% of the samples were kept. Seurat’s SCTransform was used to normalize the data and correct for mitochondrial percentage and total number of reads biases.

### Code Availability

The code is freely accessible on GitHub. CLIMB and CLIFF code can be accessed via an R library, “ClimbTheCliff,” available via the devtools command devtools::install_github(“alexdray86/ClimbTheCliff”). Documentation is available on https://github.com/alexdray86/ClimbTheCliff, and the code to reproduce the manuscript’s figures is available in https://github.com/alexdray86/Coudray2026. Package documentation, including installation instructions and worked examples, is available at https://alexdray86.github.io/ClimbTheCliff/.

## Supporting information

S1 Table Documented AML cell-subtype drug sensitivity from the literature. Documented cell-subtype drug sensitivity in Acute Myeloid Leukemia. Green signifies CLIFF’s predictions aligned with the literature (25% most sensitive/resistant). Red denotes CLIFF’s predictions conflicting with the literature (25% less sensitive/resistant). Orange represents uncertain connections, with values predicted between the 25% and 75% quartiles.
(PNG)

S1 Fig CLIMB and CLIFF framework across prediction resolutions. (A) Diagram of CLIMB/CLIFF input/output variables in overall mode. (B) Same as (A) in high-resolution mode: cell-subtype expression and cell-subtype drug sensitivity is predicted in each bulk sample. (C) Same as (A), in single-cell-resolution mode: cell-subtype expression and drug sensitivity are predicted for each single cell within each bulk sample.
(PNG)

S2 FigCLIMB 2-pass algorithm.The algorithm subsets cells in the reference single-cell matrix C two times. W represents the predicted cell-type proportions, of size N×K, N being the number of bulk samples and K the number of cell types. The first pass allows subsampling of cells in the reference matrix, which is then used during the second pass to infer the cell subtype proportions. This process is iterated twice.(PNG)

S3 Fig Robustness of CLIMB deconvolution to simulated reference-target proportion bias. (A) Illustration of the simulated pseudo-bulks with varying biases between reference and target proportions. On the right, we show the bias by comparing Van Galen et al. data with its own pseudo-bulks. (B) Accuracy, shown as a correlation coefficient (PCC), comparing true vs. predicted cell type fractions across 11 simulated scenarios for CLIMB-init, CLIMB-corrected, and Scaden methods. (C) Comparative accuracy and resilience to bias for all deconvolution methods. Resilience to bias is assumed to be equal to the variability in accuracy across different biases. (D) Same as (C), but showing the results as an error (RMSE). (E) Comparison between CLIMB-init and CLIMB-corrected deconvolution accuracy (PCC) across all analyses (in vitro and pseudo-bulks). (F) Same as (E), but showing the error instead.
(PNG)

S4 FigCross-dataset pseudo-bulk deconvolution benchmarking across cancer types.(A-D) Combined pseudo-bulk deconvolution analysis across datasets showing four accuracy metrics. (E) Pseudo-bulk deconvolution analysis across datasets (Van Galen → Naldini) displaying accuracy as Spearman correlation coefficient / R-square value per sample (one data point per sample), comparing true and predicted cell subtype proportions. (F) Deconvolution method prediction accuracy for 200 simulated pseudo-bulk samples derived from the Van Galen et al. dataset. Results presented as boxplots, per sample level, for PCC, SCC, RMSE, and R-square metrics. (G) Similar to (E), but for Naldini → Van Galen cross-dataset deconvolution analysis. (H-J) Cross-dataset deconvolution analysis in colorectal cancer, depicted as boxplots for PCC, SCC, RMSE, and R-square metrics, covering Lee → Khaliq analysis (H) and Khaliq → Lee analysis (I). (J) Average rank over 4 metrics considering Lee → Khaliq and Khaliq → Lee cross-dataset analyses. (K-M) Melanoma cross-dataset deconvolution analysis, showcasing the same metrics as (H-J), for Jerby → Tirosh analysis (K) and Tirosh → Jerby analysis (L). (N-P) Glioblastoma cross-dataset deconvolution analysis, for Neftel (10X) → Neftel (SmartSeq2) analysis (N) and Neftel (SmartSeq2) → Neftel (10X) analysis (O). (Q-S) Cross-dataset deconvolution analysis in breast cancer, for Gray → Wu analysis (Q) and Wu → Gray analysis (R).(PNG)

S5 Fig CLIMB-predicted cell-subtype proportions and running time across deconvolution methods. (A) Average cell type proportions in different categories of FAB patients. (B) Correlation (PCC) between CLIMB-predicted cell subtype proportions and LSC17 signature. (C) Correlation (PCC) between Van Galen cell type signature and cell type proportions estimated by CLIMB. (D) Correlation (PCC) between cell subtype abundance estimated by different deconvolution methods in merged BeatAML, Leucegene, TCGA-AML datasets. (E) Running time of various deconvolution methods.
(PNG)

S6 FigDeconvolution of cell subtype Expression and Simulated Over-Expression Evaluation.(A) Heatmap comparing standardized true expression from bulk RNA-seq on pure cell lines (top) with deconvoluted expression from CLIMB based on bulk RNA-seq of cell line mixes (bottom). (B) Scatter plot comparing standardized true expression (y-axis) and CLIMB’s deconvoluted expression (x-axis) from bulk RNA-seq. (C) Accuracy as PCC comparing true expression from bulk RNA-seq and deconvoluted expression from CLIMB, BayesPrism, CiberSortX (group mode, filtered and unfiltered). (D) Heatmap showing standardized true expression from pseudo-bulks (top) and CLIMB’s deconvoluted expression of pseudo-bulks (bottom). (E) Scatter plot of deconvoluted expression (x-axis) versus true expression (y-axis) for pseudo-bulks. (F) Accuracy as PCC comparing true expression from bulk RNA-seq and deconvoluted expression from CLIMB, BayesPrism, CiberSortX (group mode, filtered and unfiltered). (G) Schematic of cross-dataset analysis with over-expression of 20 genes in 7/13 bulk samples and in HSC-like cell subtype. (H) Fold changes observed at bulk and cell-subtype levels when inducing single cells with a fold change of 10 before pseudo-bulk generation. Control genes are also compared. (I) Comparison between true and CLIMB’s deconvoluted expression for HSC-like with over-expressed genes. Top heatmap displays true expression, with 20 over-expressed genes (right) and 20 random non-over-expressed genes (left). Overexpression samples are above, WT samples below. Bottom heatmap shows CLIMB’s deconvoluted expression with the same gene and sample order. (J) Accuracy to detect correct over-expressed genes in HSC-like, using $-\log_{10}(padj)\cdot$ FC by DESeq2, presented as an average AUC-ROC over 20 genes. (K) Similar to (J), but showing accuracy to detect the correct cell subtype among cancer cell subtypes for each over-expressed gene, averaged over 20 genes. (L) Accuracy as PCC comparing true expression with CLIMB, BayesPrism, and CiberSortX deconvoluted expression.(PNG)

S7 Fig CLIFF prediction accuracy and robustness across input parameters. (A) Comparative analysis of true bulk viability (measured for 3 timepoints, 7 drugs, and 10 doses) against reconstructed bulk viability, using experimentally-assessed viability of pure cell lines comprising the bulks. (B) True cell line drug sensitivity for multiple timepoints, cell lines, and drugs, juxtaposed with CLIMB’s high-resolution mode predictions (subsequently averaged at cell type level). (C) CLIFF’s predictive accuracy presented as Pearson correlation coefficient (PCC), grouped by cell lines. (D) Accuracy comparison of CLIFF with varying numbers of input genes, based on the 200 simulated bulks. We evaluated four modes for CLIFF: CLIFF-overall, CLIFF-overall-L2 (CLIFF-overall with additional L2 regularization), CLIFF-highres (subsequently average at cell subtype level), and CLIFF-highres-L2 (CLIFF-highres with L2 regularization). (E) Predictive accuracy depicted as PCC for deconvolution of cell subtype proportions in 200 simulated pseudo-bulks, featuring CLIFF-overall, CLIFF-highres, Karakaslar’s approach (linear regression using MuSiC-derived proportions as input matrix), and Bottomly’s approach (correlation between cell-subtype score obtained from PC1 of cell subtype signature, and bulk drug sensitivity). (F) Simulated mutational landscape for 200 simulated bulks employed in high-resolution drug sensitivity deconvolution.
(PNG)

S8 Fig CLIFF-predicted drug specificity and mutation associations in the beatAML cohort. (A) Scatter plot illustrating the Pearson correlation coefficient (PCC) between Bottomly’s drug sensitivity predictions (for the 6 cancer cell subtypes defined by Van Galen et al.) and standardized CLIFF-overall predicted AUC (relative values of cell subtype drug sensitivity). Each data point represents a specific cell subtype-drug combination. (B) Corresponding to (A), this plot displays the overall PCC (averaging 6 data points for the 6 cancer cell subtypes) for the 14 drugs listed in S1 Table. (C) Predicted cancer cell specificity for drugs within the beatAML cohort, as projected by CLIFF-overall. (D) Forecasted drug specificity for undifferentiated versus differentiated blasts in the beatAML cohort, according to CLIFF-overall. (E) Overall correlations between mutations and cell subtype proportions using CLIMB’s deconvoluted proportions on the beatAML cohort. A set of mutations with at least 5 positive samples is analyzed, with size-effect/significance depicted through t-test-derived measures. Positive correlations are represented in red, negative in blue. The size indicates the size effect (mean proportion difference). (F) Correlation between high-resolution deconvoluted drug sensitivity for each cell subtype and mutations in the beatAML cohort, focusing on the A-674563 compound. (G) Same as (F), but for the venetoclax drug.
(PNG)

S9 Fig Deconvolution accuracy validation using in vitro cell-line mixtures and nasal-swab pseudo-bulks. (A) Accuracy metrics for deconvolution of pseudo-bulk samples derived from an in vitro cell-line mixture experiment generated from scRNA-seq, deconvoluted using CLIMB, NNLS (using a signature matrix), and NNLS implemented in the MuSiC R library (NNLS-MuSiC). (B) Accuracy metrics for deconvolution of real bulk RNA-seq samples derived from an in vitro cell-line mixture experiment, deconvoluted using CLIMB, NNLS (using a signature matrix), and NNLS implemented in the MuSiC R library (NNLS-MuSiC). (C) Spearman correlation between pseudo-bulk (left) or real bulk RNA-seq samples (right) and the average expression of each of the four cell types contained in the mixtures. (D) Correlation of average normalized expression between pseudo-bulk and real bulk RNA-seq samples containing the same cell-type proportions. (E) Accuracy metrics for deconvolution of 29 nasal-swab pseudo-bulk samples from COVID-19 and healthy patients, using 29 foreign samples as the reference.
(PNG)

S10 FigAblation of CLIMB’s accuracy-driving components and robustness characterization of CLIFF.(A) CLIMB accuracy before (props.init) and after (props.corrected) empirical-Bayes correction vs. NNLS, across 11 ground-truth datasets, isolating the respective contributions of iterative reweighting (props.init vs. props.corrected) and cell-level resolution (CLIMB vs. NNLS, which uses averaged cell-type profiles). (**B**) Accuracy vs. reference coverage (5–500 cells/type) on a non-hematopoietic dataset: props.init improves with coverage while NNLS stays flat. (**C**) CLIFF identifiability: sensitivity recovery error vs. cross-sample proportion correlation (ρ). (**D**) EM initialization sensitivity: first-iteration vs. converged error for 25 random restarts at ρ=0.5,0.9,0.99; dashed crosshairs mark the default (zero) initialization. (**E**) Converged error across the initialization landscape (starting mean vs. spread) at ρ=0.9; outlined cell marks the default. (**F**) climb()/cliff() runtime vs. sample count, gene count, cell-type count, and reference size. (**G**) Propagation of calibrated proportion noise (matched to CLIMB-corrected, CLIMB-init, and NNLS accuracy) into CLIFF sensitivity error.(PNG)
