# Supplementary material for: Computational deconvolution of drug sensitivity via single-cell–to–bulk transcriptome mapping
Source: PLoS Comput Biol. 2026 Oct 16;22(10):e1014862. doi: 10.1371/journal.pcbi.1014862 (PMC13712728; doi:10.1371/journal.pcbi.1014862)
Supplement: Supplementary file 1 [file pcbi.1014862.s012.pdf]

Response to Reviewers

We thank the editor for its feedback. We have carefully revised the manuscript, figures, and supporting materials to address each point. Below we provide a point-by-point response.

1. Title and manuscript focus
   Reviewer comment: Consider slight reformulation of the title and manuscript to emphasize methodological advances and broaden readership. Show applicability more generally, framing the manuscript as a methodological paper with AML as an application.

Response:
We have revised the title to: "*Computational deconvolution of drug sensitivity via single-cell–to–bulk transcriptome mapping.*" Furthermore, we reframed the Abstract, Introduction, parts of the Results, and Discussion to emphasize the general utility of CLIMB and CLIFF as tools for bulk-to-single-cell deconvolution in any context where paired bulk RNA-seq and functional measurements are available. AML is now presented as a primary application example, without limiting the broader applicability of the methods. Additionally, we included a dataset with COVID-19 and healthy nasopharynx samples to demonstrate the broader applicability of our framework.

2. Figure 1 revisions
Reviewer comment: Revise Figure 1 to include all key methodological points.

Response:
We have updated Figure 1 to:
• Clearly indicate that reference scRNA-seq and bulk RNA-seq can come from independent samples (Fig1A).
• Include the optional Empirical Bayes sub-sampling step used to mitigate extreme reference composition bias (Fig1A).
• Show all input data used by CLIFF (Fig1C).
• Represent the EM algorithm in CLIFF and indicate iterative updates (Fig1B).
• Clarify that CLIFF runs on top of CLIMB outputs (Fig1C).

Figure legend and schematic arrows now explicitly indicate the flow from CLIMB to CLIFF, including both inputs and learned variables (Fig1C).

3. Additional applications
Reviewer comment: If possible, please add some additional application to show the methods are generally applicable to other cancers/diseases.

Response:
We included a dataset with COVID-19 and healthy nasopharynx samples to demonstrate the broader applicability of our framework.

4. NNLS performance investigation
Reviewer comment: f possible, please fully understand why NNLS can perform so bad as Random. Obviously, NNLS uses information (e.g. signature matrix) from data, and should be better than Random.

Response:
We conducted additional analyses comparing two implementations of NNLS with CLIMB on both pseudo-bulk and real bulk RNA-seq datasets. We now report that NNLS performs well when pseudo-bulk and reference scRNA-seq are derived from the same technology, but its accuracy drops dramatically on real bulk RNA-seq. We added text explaining this sensitivity as due to cross-platform discrepancies, reference composition bias, and reliance on averaged cell-type expression, which are mitigated by CLIMB's iterative, cell-resolved framework (see revised Results, SFig. 9).

5. Data and code availability
Reviewer comment: Make all the data and codes truly available for reproducibility.

Response:
We have updated our repositories to provide public access, a R library that can be easily installed with its full documentation, and anther repository containing notebook to reproduce all manuscript's figures:

- Documentation is available on https://github.com/alexdray86/ClimbTheCliff
- The code to reproduce the manuscript's figures is available in https://github.com/alexdray86/Coudray2026

These repositories now include:

- All code to reproduce each figure in the manuscript.
- Simulation data used in benchmarking, along with real datasets where permitted.
- A detailed README.md with instructions for reproducing the analyses.

We believe these revisions address all editor's concerns, clarify the general applicability of our methods, and ensure reproducibility. We thank the editor again for its helpful suggestions.
