# Supplementary material for: Computational deconvolution of drug sensitivity via single-cell–to–bulk transcriptome mapping
Source: PLoS Comput Biol. 2026 Oct 16;22(10):e1014862. doi: 10.1371/journal.pcbi.1014862 (PMC13712728; doi:10.1371/journal.pcbi.1014862)
Supplement: Supplementary file 2 [file pcbi.1014862.s013.pdf]

Answer to reviewers

PCOMPBIOL-D-25-01865R2
Computational deconvolution of drug sensitivity via single-cell–to–bulk transcriptome mapping
PLOS Computational Biology

Reviewer #1: This is a well-revised manuscript that introduces a valuable computational tool for the integration of single-cell transcriptomics and bulk functional assays. The authors have adequately addressed previous critiques and demonstrated both accuracy and generality. With minor clarifications and editorial polish, the paper will be suitable for publication in PLOS Computational Biology.

[...]
Weaknesses:
1. Clarity of the Core Innovation: While the performance is strong, the manuscript could more clearly articulate what specific component of CLIMB's "iterative, cell-resolved framework" is primarily responsible for its robustness to cross-platform noise compared to other non-DL methods. Is it the iterative reweighting, the cell-level resolution, or a combination? A more focused ablation study would strengthen the claims.

To answer this, we compared CLIMB's error before (props.init) and after iterative weighting (props.corrected) to NNLS (averaged reference profile). Since CLIMB uses a linear model with a non-negative coefficient constraint, it is close to a single-cell-level NNLS model. The advantage from single-cell resolution can therefore be derived, roughly, by comparing props.init to NNLS, while the advantage from iterative weighting is shown by comparing props.init to props.corrected.

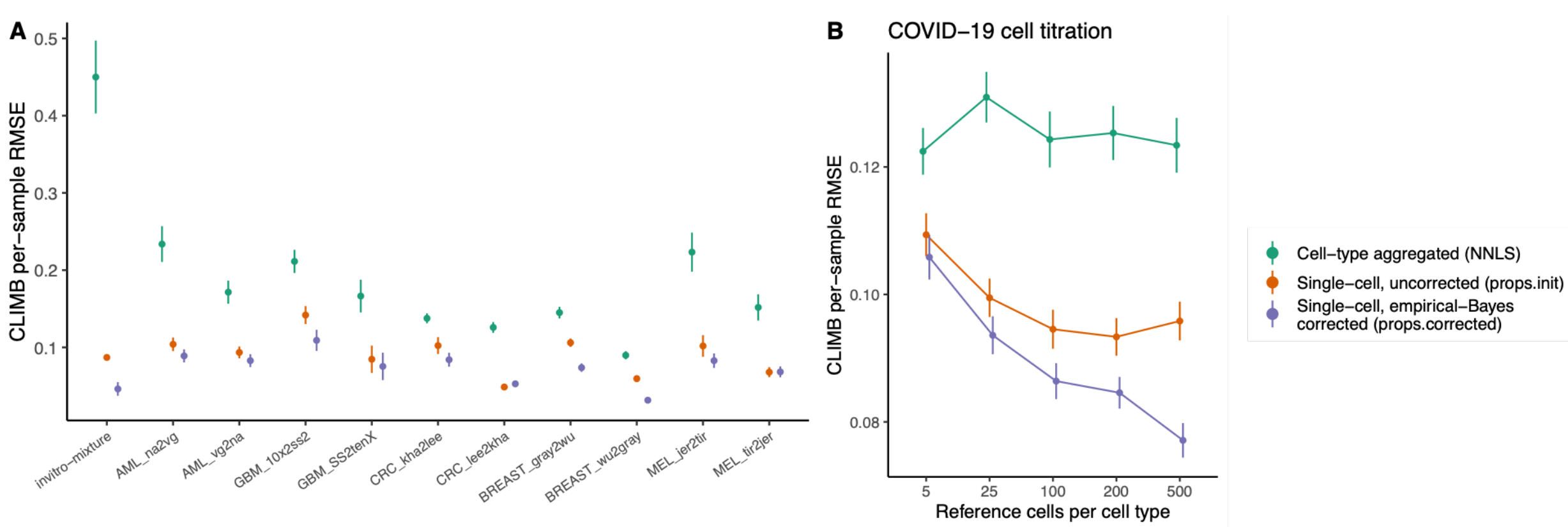

Comparing props.init to props.corrected across 11 ground-truth datasets (6 in-vitro mixtures, 10 cross-dataset pairs), the empirical-Bayes correction improved accuracy in 9/11 (mean RMSE 0.076 vs. 0.095), extending the result on p.7/Supplemental Fig. 3E-F to a larger, more systematic scale (new Supplemental Figure 10A).

Isolating cell-level resolution, we compared CLIMB's props.init (no iterative reweighting, for a clean single-cell-resolution-vs-NNLS comparison) to the manuscript's NNLS baseline (Methods Eq. 1, averaged reference profile) on a non-hematopoietic dataset (COVID-19 nasal-swab), subsampling

the reference from 5 to 500 cells/type. NNLS stayed flat (RMSE 0.123-0.131) — structurally unable to use additional cells — while props.init improved with coverage (0.109 to 0.096; new Supplemental Figure 10B).

Both iterative weighting and single-cell resolution therefore benefit CLIMB, though the single-cell-resolution advantage appears larger.

We have added the Supplemental Figure 10 A-B to the manuscript, together with its description in the Result section, and discussion about CLIMB's cell-level resolution versus iterative framework advantages.

2. Abstract clarity: The abstract could better highlight the methodological advance (cell-level drug sensitivity deconvolution) before diving into results. Suggest to restructure as: "We introduce CLIMB/CLIFF, a framework that... We first validate... We then apply... Our approach..."

We thank the reviewer for this suggestion. We have restructured the abstract along the proposed skeleton: it is now naming CLIMB and CLIFF and stating the two-step framework's methodological advance — cell-type and single-cell-resolved drug sensitivity deconvolution — before any results, then presents validation (in-vitro cell line mixtures with known ground truth, extended to cross-dataset and pseudo-bulk analyses) ahead of the beatAML application and findings.

3. Technical accessibility: Some methodological descriptions (e.g., EM algorithm, sigmoid linking expression to drug sensitivity) remain dense. Please add a brief conceptual summary in the main text (or a schematic box) explaining how CLIFF's probabilistic model works intuitively.

We thank the reviewer for this suggestion. We have added an intuitive, plain-language paragraph at the opening of the "CLIFF - Cell fate inference for drug sensitivity deconvolution" Methods subsection, before the formal derivation, explaining how the model treats each observed bulk drug response as a mixture of hidden cell-subtype-specific responses, how the Expectation-Maximization algorithm's E-step and M-step alternate to resolve this mixture, and what role the sigmoid link plays — with a cross-reference back to the existing schematic in Fig. 1B.

4. Figure and supplementary referencing: Some references to supplementary figures are incomplete or misnumbered (e.g., "SFig.??" on p. 72). Occasional formatting inconsistencies and some references are truncated or incomplete (e.g., p. 71, reference to "BisqueRNA [40]" but reference list shows [36]).

We thank the reviewer for catching these inconsistencies. We have corrected the two broken references cited by the reviewers. This also prompted us to perform a full cross-check of every in-text citation number against the reference list, rather than only the instance the reviewer flagged. This check surfaced one additional, previously unnoticed mismatch — a citation to Zeng et al. that

pointed to the wrong reference number in the bibliography — which we have now corrected as well. We are confident the reference and figure numbering is now consistent throughout the manuscript.

5. The discussion of limitations is appropriate but could be expanded regarding potential biases from scRNA-seq reference selection or platform effects. Briefly note how reference quality/coverage might affect CLIMB's performance in non-hematopoietic contexts.

We thank the reviewer for this point. CLIMB's iterative Empirical Bayes sub-sampling is specifically designed to correct for cell types that are present in the reference but at a proportion that does not match their true abundance in the target bulk sample — SFig. 3 quantifies this correction across eleven levels of reference-to-bulk proportion mismatch. We agree with the reviewer, however, that this correction has an inherent limit: it can only reweight cells that were captured in the reference in the first place, and cannot recover a cell type that is absent or severely depleted from the reference altogether. This is a real concern specifically for non-hematopoietic (solid) tissues, which require enzymatic or mechanical dissociation prior to single-cell profiling — unlike blood or bone marrow, where cells are naturally in suspension — and dissociation is known to deplete certain cell types systematically rather than at random, and to introduce stress-response transcriptional artifacts into surviving cells. We have added a paragraph to the Discussion (Limitations) making this distinction explicit and citing the relevant literature.

Reviewer #2: This paper proposes a two-step framework, CLIMB–CLIFF, to infer cell-subtype composition, expression, and drug sensitivity from bulk RNA-seq and bulk drug response data by leveraging reference scRNA-seq. CLIMB performs bulk-to-single-cell deconvolution at the single-cell level, correcting reference-induced bias via empirical Bayes sub-sampling, while CLIFF models bulk drug survival as a mixture of latent subtype-specific survival processes and infers cell-type–specific drug sensitivities through an EM framework. The work addresses a relevant and challenging problem—recovering cell-type–resolved drug response from bulk assays—and provides a probabilistic formulation that explicitly models latent cellular heterogeneity rather than relying on direct regression. Here are my comments:

1. The inference of cell-subtype–specific drug sensitivities relies on variation in cell-subtype proportions across samples. When subtype proportions are highly correlated or show limited variability, the mixture model may suffer from identifiability issues, potentially leading to unstable or non-unique estimates of subtype-specific sensitivities. An explicit discussion of identifiability conditions or sensitivity analyses under correlated subtype proportions would further strengthen the framework.

We thank the reviewer for raising this important point regarding identifiability. CLIFF's per-cell-subtype parameters are identifiable only if the sample-by-cell-subtype proportion matrix has full column rank; short of that, high cross-sample correlation behaves as multicollinearity, and a cell subtype with near-constant proportion across samples contributes negligible rank regardless of its

correlation with other subtypes — both are instances of the same underlying condition. To characterize this empirically, we performed a dedicated simulation with four cell subtypes of known drug sensitivities (using our in-vitro experiment as a starting point), generating proportions with a tunable cross-sample correlation ρ and feeding them into the unmodified cliff() function. Recovery remained stable for ρ up to approximately 0.8 (RMSE 0.15–0.17), degraded progressively from ρ=0.9 (RMSE 0.18), and sharply by ρ=0.99 (RMSE 0.26), at which point all four recovered cell-type sensitivities converged to within 0.02 of one another despite spanning true values from 0.15 to 0.85 — a clear identifiability breakdown as anticipated by the reviewer. We have added this identifiability condition, the simulation, and practical guidance for users to the Discussion (Supplemental Figure 10C). We are grateful to the reviewer for pointing us toward this analysis, which we believe meaningfully strengthens the manuscript.

2. The EM-based optimization involves multiple layers of latent variables and nonlinear parameterization. It is unclear how sensitive the results are to initialization of π or β, or whether multiple local optima exist. The manuscript would benefit from a more thorough analysis of EM convergence properties, including sensitivity to initialization and potential local optima.

We thank the reviewer for raising this important point about the robustness of the EM-based optimization underlying CLIFF. Since π is directly derived from β, we benchmark hereafter distinct initialization for β: the following conclusion should translate for π initialization as well, since they are only linked by a sigmoid function call.

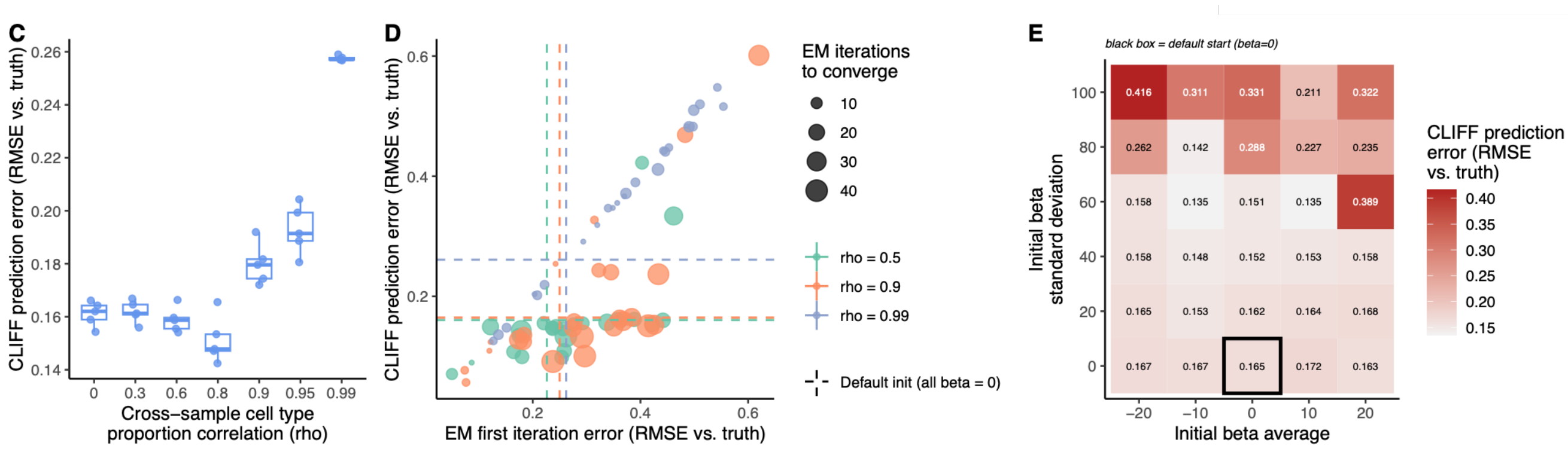

To assess sensitivity to initialization, we used a modified version of cliff() that accepts an arbitrary starting coefficient vector (the shipped default is a vector of zeros) and ran 25 random restarts at three correlation levels (rho = 0.5, 0.9, and 0.99), spanning the well- to poorly-identified regimes from our response to Point 1. Restarts converged to a distinctly worse optimum in a minority of cases under good identifiability (2/25 at rho=0.5; 7/25 at rho=0.9) and in a majority of cases under high inter-cell-type proportion correlation condition (19/25 at rho=0.99), confirming that multiple local optima exist and that they become more common as identifiability worsens (Supplemental Figure 10D). The default initialization performed like a typical restart (dashed lines) rather than an unusually good one.

We also swept the mean and spread of the starting coefficients over a much wider range (Supplemental Figure 10E) and found accuracy is less sensitive to the mean than to the spread, degrading once the spread becomes large. Across this wider range, the default (zero) initialization used throughout the manuscript remained on the favorable side, confirming that our results are not an artifact of an unusually favorable or unfavorable starting point and that CLIFF's estimates are reasonably robust to initialization. We have added a point to the Discussion on this, on top of the new results shown in Supplemental Figure 10.

3. How is the performance of CLIMB–CLIFF on large datasets? It's better to provide computational complexity or runtime benchmarks.

We thank the reviewer for this suggestion; a runtime benchmark is indeed a useful addition, and we have included one as a new panel in Supplemental Figure 10.

We benchmarked climb() and cliff() while varying the number of bulk samples (10 to 100), genes (200 to 2,000), reference cell types (3 to 15), and total reference cells (up to 15,000, matching the scale of the manuscript's AML reference). climb() scales roughly linearly with reference size and dominates overall runtime, ranging from 2.9 to 13.9 seconds per bulk sample as the reference grows from 1,000 to 15,000 cells (Supplemental Figure 10F). cliff() scales with the number of bulk samples but is largely insensitive to reference size. Combined, a full CLIMB-CLIFF run on 100 samples against a 15,000-cell reference completed in just above 2 minutes (128s), confirming that both methods remain practical at the scale of the datasets used in this study and well beyond it.

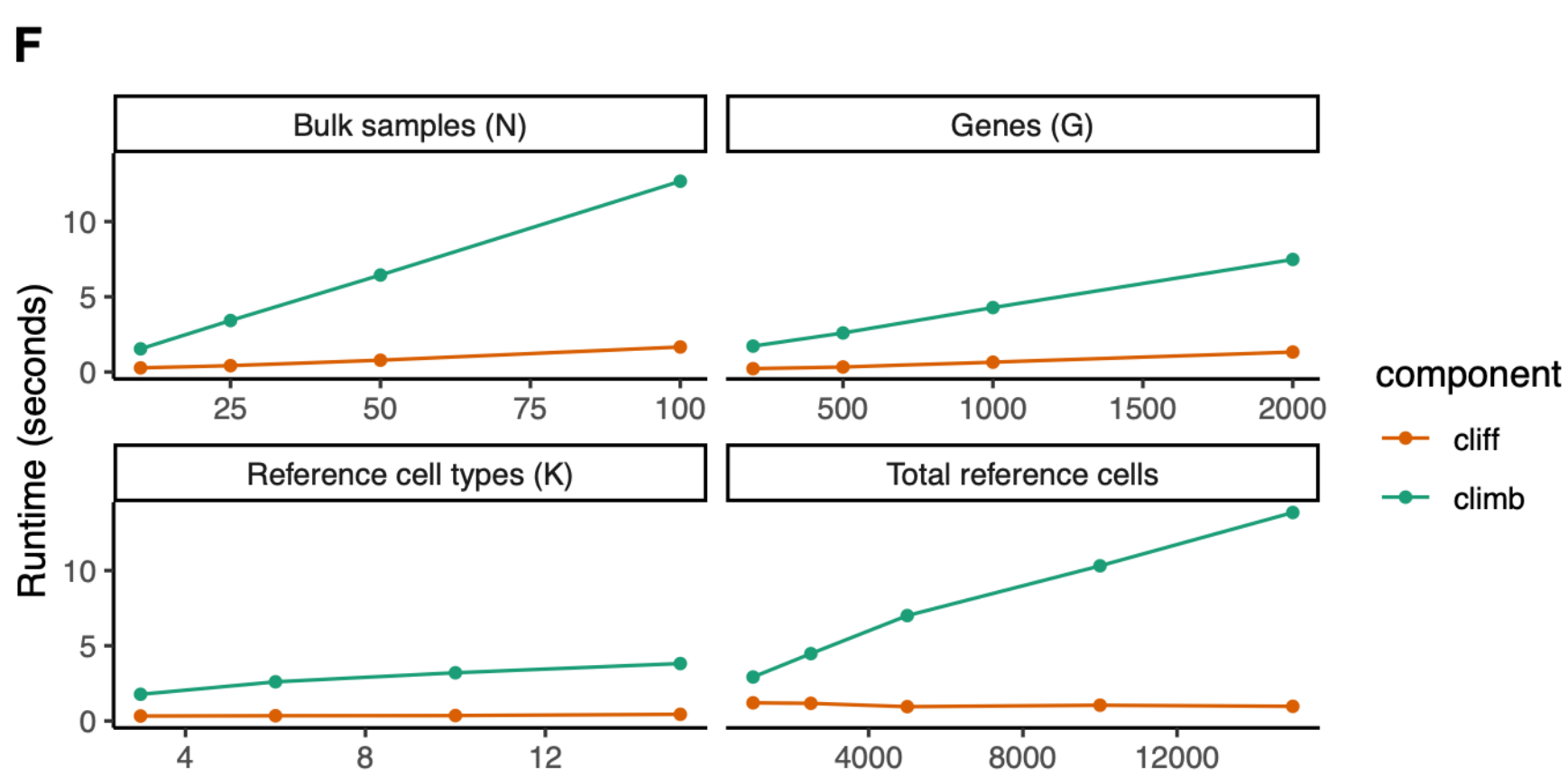

4. Please clarify how uncertainty in cell-type proportion estimates from CLIMB propagates into the inference of cell-type–specific drug sensitivities in CLIFF.

We thank the reviewer for this question, which addresses an important aspect of how uncertainty compounds across the two stages of our pipeline.

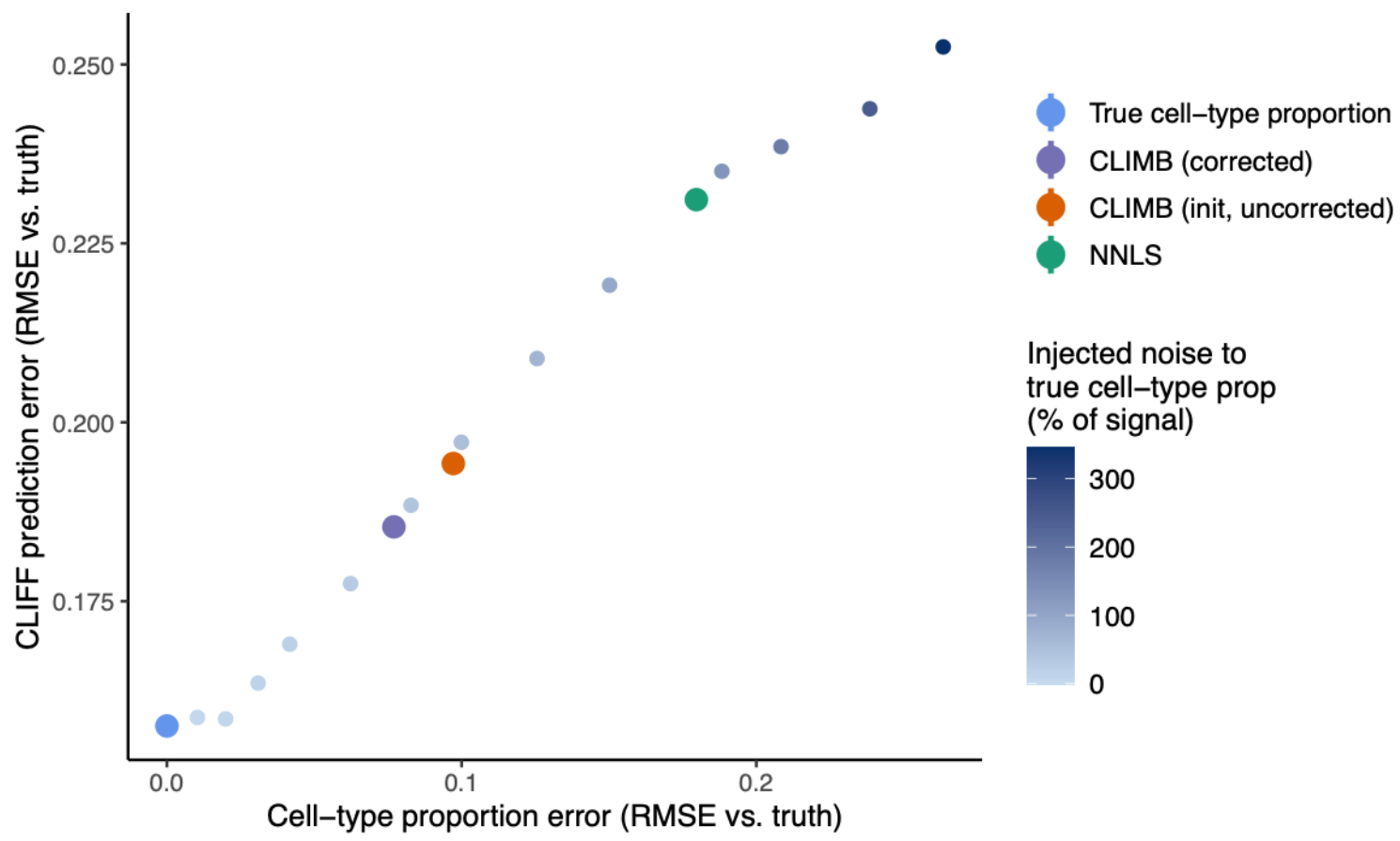

To quantify this directly, we injected controlled, calibrated levels of proportion error, matched to the actual accuracy achieved by true proportions, CLIMB-corrected estimates, CLIMB-uncorrected estimates, and NNLS, into a well-identified simulation, and measured the resulting error in CLIFF's cell-type-specific drug sensitivity predictions. The relationship is monotonic: RMSE rises from 0.158 with true proportions, to 0.185 with CLIMB-corrected estimates, to 0.194 with CLIMB-uncorrected estimates, and to 0.231 with NNLS estimates. Error does not fall to zero even when proportions are known exactly, reflecting an inherent measurement-noise floor in the drug-sensitivity data itself. Notably, the roughly two-fold difference in proportion accuracy between CLIMB-corrected and NNLS translates into a more modest ~25% difference in downstream sensitivity error, indicating that CLIFF does not seem to amplify upstream proportion uncertainty (Supplemental Figure 10G, above). We have added a short discussion of this point to the manuscript.
