# Supplementary material for: Computational deconvolution of drug sensitivity via single-cell–to–bulk transcriptome mapping
Source: PLoS Comput Biol. 2026 Oct 16;22(10):e1014862. doi: 10.1371/journal.pcbi.1014862 (PMC13712728; doi:10.1371/journal.pcbi.1014862)
Supplement: Peer review history

# Editor report: Decision Letter 0

**DOI:** 10.1371/journal.pcbi.1014862.r001

**Authors:** Ferhat Ay, Zhaoyuan Fang

2 Nov 2025

PCOMPBIOL-D-25-01865

Profiling drug sensitivity of leukemic stem cells via bulk-to-single-cell deconvolution

PLOS Computational Biology

Dear Dr. Coudray,

Thank you for submitting your manuscript to PLOS Computational Biology. After careful consideration, we feel that it has merit but does not fully meet PLOS Computational Biology's publication criteria as it currently stands. Therefore, we invite you to submit a revised version of the manuscript that addresses the points raised during the review process.

Please submit your revised manuscript within 30 days Jan 02 2026 11:59PM. If you will need more time than this to complete your revisions, please reply to this message or contact the journal office at ploscompbiol@plos.org. When you're ready to submit your revision, log on to https://www.editorialmanager.com/pcompbiol/ and select the 'Submissions Needing Revision' folder to locate your manuscript file.

Please include the following items when submitting your revised manuscript:

* A rebuttal letter that responds to each point raised by the editor and reviewer(s). You should upload this letter as a separate file labeled 'Response to Reviewers'. This file does not need to include responses to formatting updates and technical items listed in the 'Journal Requirements' section below.

* A marked-up copy of your manuscript that highlights changes made to the original version. You should upload this as a separate file labeled 'Revised Manuscript with Track Changes'.

* An unmarked version of your revised paper without tracked changes. You should upload this as a separate file labeled 'Manuscript'.

If you would like to make changes to your financial disclosure, competing interests statement, or data availability statement, please make these updates within the submission form at the time of resubmission. Guidelines for resubmitting your figure files are available below the reviewer comments at the end of this letter.

We look forward to receiving your revised manuscript.

Kind regards,

Zhaoyuan Fang

Academic Editor

PLOS Computational Biology

Ferhat Ay

Section Editor

PLOS Computational Biology

**Editor Comments :**

We can see it is a valuable work with interesting methodological progress on deconvolution bulk data matrix (CLIMB) as well as drug responses data (CLIFF), and with useful applications to leukemia. During the three rounds of revisions in Nature Communications, the authors have made considerable efforts to consolidate their models, to reduce potential biases, and to strength benchmarking designs. Therefore, we recommend the authors to revise on just a few persisting issues, rather than being sent out for a new reviewing process.

1. Consier slight reformulation of the title and manuscript to emphasize the methodological advances and also to gain a wider readership.

The authors are suggested to show its applicability in a more general sense, so that it could benefit more researchers in the biomedical fields. For the title, you can try something like "A bulk-to-single-cell deconvolution approach for drug sensitivity in leukemia". For the manuscript, you can reframe it from Introduction through Discussion to make it a more typical methodological paper with applications to AML.

2. Revise Figure 1 to include all key methodological points.

For instance: Make clear whether reference sc and bulk datasets should come from the same sample(s); Add the optional sub-sampling step for removing biases in extreme cases of imbalanced cell type compositions; Add all input data for CLIFF; Add EM algorithm in CLIFF; Clarify that CLIFF runs on top of CLIMB; Any other additional points.

3. If possible, please add some additional application to show the methods are generally applicable to other cancers/diseases.

4. If possible, please fully understand why NNLS can perform so bad as Random.

Obviously, NNLS uses information (e.g. signature matrix) from data, and should be better than Random.

5. Make all the data and codes truly available for reproducibility.

The whole study (including each figure) should be reproducible by other researchers, which is important to benefit the community truly. As of now, the claimed repositories can not be found on github: "https://alexdray86.github.io/CLIMB/build/index.html and https://alexdray86.github.io/CLIFF/build/index.html website", which should be made available permantly. In addition, the simulation data and codes should also be made publicly accessible with sufficient description in a README.md file on github.

**Journal Requirements:**

1) Please ensure that the CRediT author contributions listed for every co-author are completed accurately and in full.

At this stage, the following Authors/Authors require contributions: Alexandre Coudray, Romain Forey, Benjamin Bejar Haho, Filipe Martins, Johana CARLEVARO-FITA, SHAOLINE SHEPPARD, Sandra Offner, Guillaume Obozinski, Gioele La Manno, and Dider Trono. Please ensure that the full contributions of each author are acknowledged in the "Add/Edit/Remove Authors" section of our submission form.

The list of CRediT author contributions may be found here: https://journals.plos.org/ploscompbiol/s/authorship#loc-author-contributions

2) We ask that a manuscript source file is provided at Revision. Please upload your manuscript file as a .doc, .docx, .rtf or .tex. If you are providing a .tex file, please upload it under the item type u2018LaTeX Source Fileu2019 and leave your .pdf version as the item type u2018Manuscriptu2019.

3) Please provide an Author Summary. This should appear in your manuscript between the Abstract (if applicable) and the Introduction, and should be 150-200 words long. The aim should be to make your findings accessible to a wide audience that includes both scientists and non-scientists. Sample summaries can be found on our website under Submission Guidelines:

https://journals.plos.org/ploscompbiol/s/submission-guidelines#loc-parts-of-a-submission

4) We do not publish any copyright or trademark symbols that usually accompany proprietary names, eg ©,  ®, or TM  (e.g. next to drug or reagent names). Therefore please remove all instances of trademark/copyright symbols throughout the text, including:

- TM on pages: 27, and 28.

5) Your manuscript is missing the following section heading: Abstract.  Please ensure all required sections are present and in the correct order. Make sure section heading levels are clearly indicated in the manuscript text, and limit sub-sections to 3 heading levels. An outline of the required sections can be consulted in our submission guidelines here:

https://journals.plos.org/ploscompbiol/s/submission-guidelines#loc-parts-of-a-submission

6) Please upload all main figures as separate Figure files in .tif or .eps format. For more information about how to convert and format your figure files please see our guidelines:

https://journals.plos.org/ploscompbiol/s/figures

7) We notice that your supplementary Figures, and Table are included in the manuscript file. Please remove them and upload them with the file type 'Supporting Information'. Please ensure that each Supporting Information file has a legend listed in the manuscript after the references list.

8) Some material included in your submission may be copyrighted. According to PLOSu2019s copyright policy, authors who use figures or other material (e.g., graphics, clipart, maps) from another author or copyright holder must demonstrate or obtain permission to publish this material under the Creative Commons Attribution 4.0 International (CC BY 4.0) License used by PLOS journals. Please closely review the details of PLOSu2019s copyright requirements here: PLOS Licenses and Copyright. If you need to request permissions from a copyright holder, you may use PLOS's Copyright Content Permission form.

Please respond directly to this email and provide any known details concerning your material's license terms and permissions required for reuse, even if you have not yet obtained copyright permissions or are unsure of your material's copyright compatibility. Once you have responded and addressed all other outstanding technical requirements, you may resubmit your manuscript within Editorial Manager.

Potential Copyright Issues:

i) Figures 2A, 3A, 4A, and S6G. Please confirm whether you drew the images / clip-art within the figure panels by hand. If you did not draw the images, please provide (a) a link to the source of the images or icons and their license / terms of use; or (b) written permission from the copyright holder to publish the images or icons under our CC BY 4.0 license. Alternatively, you may replace the images with open source alternatives. See these open source resources you may use to replace images / clip-art:

- https://commons.wikimedia.org

- https://openclipart.org/.

9) Please update your Data Availability Statement in the online submission form to include a direct link to access the code.

10) Please amend your detailed Financial Disclosure statement. This is published with the article. It must therefore be completed in full sentences and contain the exact wording you wish to be published.

1) State the initials, alongside each funding source, of each author to receive each grant. For example: "This work was supported by the National Institutes of Health (####### to AM; ###### to CJ) and the National Science Foundation (###### to AM)."

2) State what role the funders took in the study. If the funders had no role in your study, please state: "The funders had no role in study design, data collection and analysis, decision to publish, or preparation of the manuscript."

11) Please ensure that the funders and grant numbers match between the Financial Disclosure field and the Funding Information tab in your submission form. Note that the funders must be provided in the same order in both places as well.

12) Please ensure that the Author List in your manuscript file matches the Author List in the online submission form exactly. Authors should be listed in the same order, and the order of individual first and last author names must be identical in both locations.

Note: If the reviewer comments include a recommendation to cite specific previously published works, please review and evaluate these publications to determine whether they are relevant and should be cited. There is no requirement to cite these works unless the editor has indicated otherwise.

**Figure resubmission:**

**Reproducibility:**

To enhance the reproducibility of your results, we recommend that authors of applicable studies deposit laboratory protocols in protocols.io, where a protocol can be assigned its own identifier (DOI) such that it can be cited independently in the future. Additionally, PLOS ONE offers an option to publish peer-reviewed clinical study protocols. Read more information on sharing protocols at https://plos.org/protocols?utm_medium=editorial-email&utm_source=authorletters&utm_campaign=protocols

# Author comment: Author response to Decision Letter 1

**DOI:** 10.1371/journal.pcbi.1014862.r002

31 Dec 2025

Attachment Submitted filename: ResponseToReviewers.pdf

# Peer review: Decision Letter 1

**DOI:** 10.1371/journal.pcbi.1014862.r003

**Authors:** Ferhat Ay, Zhaoyuan Fang

27 Feb 2026

PCOMPBIOL-D-25-01865R1

Computational deconvolution of drug sensitivity via single-cell–to–bulk transcriptome mapping

PLOS Computational Biology

Dear Dr. Coudray,

Thank you for submitting your manuscript to PLOS Computational Biology. After careful consideration, we feel that it has merit but does not fully meet PLOS Computational Biology's publication criteria as it currently stands. Therefore, we invite you to submit a revised version of the manuscript that addresses the points raised during the review process.

Please submit your revised manuscript by Apr 29 2026 11:59PM. If you will need more time than this to complete your revisions, please reply to this message or contact the journal office at ploscompbiol@plos.org. When you're ready to submit your revision, log on to https://www.editorialmanager.com/pcompbiol/ and select the 'Submissions Needing Revision' folder to locate your manuscript file.

Please include the following items when submitting your revised manuscript:

* A letter that responds to each point raised by the editor and reviewer(s). You should upload this letter as a separate file labeled 'Response to Reviewers'. This file does not need to include responses to formatting updates and technical items listed in the 'Journal Requirements' section below.

* A marked-up copy of your manuscript that highlights changes made to the original version. You should upload this as a separate file labeled 'Revised Manuscript with Track Changes'.

* An unmarked version of your revised paper without tracked changes. You should upload this as a separate file labeled 'Manuscript'.

If you would like to make changes to your financial disclosure, competing interests statement, or data availability statement, please make these updates within the submission form at the time of resubmission. Guidelines for resubmitting your figure files are available below the reviewer comments at the end of this letter

We look forward to receiving your revised manuscript.

Kind regards,

Zhaoyuan Fang

Academic Editor

PLOS Computational Biology

Ferhat Ay

Section Editor

PLOS Computational Biology

**Journal Requirements:**

If the reviewer comments include a recommendation to cite specific previously published works, please review and evaluate these publications to determine whether they are relevant and should be cited. There is no requirement to cite these works unless the editor has indicated otherwise.

1) We have noticed that you have uploaded Supporting Information files, but you have not included a list of legends. Please add a full list of legends for your Supporting Information files after the references list.

2)  Please ensure that the files are uploaded in the online submission form in a correct numerical order.

3) Please amend your detailed Financial Disclosure statement. This is published with the article. It must therefore be completed in full sentences and contain the exact wording you wish to be published.

1. Please clarify all sources of funding (financial or material support) for your study. List the grants (with grant number) or organizations (with url) that supported your study, including funding received from your institution.

2. State the initials, alongside each funding source, of each author to receive each grant.

3. State what role the funders took in the study. If the funders had no role in your study, please state: “The funders had no role in study design, data collection and analysis, decision to publish, or preparation of the manuscript.”

4. If any authors received a salary from any of your funders, please state which authors and which funders.

**Reviewers' comments:**

Reviewer's Responses to Questions

**Comments to the Authors:**

**Please note here if the review is uploaded as an attachment.**

Reviewer #1: This is a well-revised manuscript that introduces a valuable computational tool for the integration of single-cell transcriptomics and bulk functional assays. The authors have adequately addressed previous critiques and demonstrated both accuracy and generality. With minor clarifications and editorial polish, the paper will be suitable for publication in PLOS Computational Biology.

Strengths

1. Methodological innovation – The two-step framework (CLIMB for cellular deconvolution, CLIFF for drug sensitivity deconvolution) is novel, well-motivated, and rigorously validated.

2. Comprehensive benchmarking – The authors compare CLIMB/CLIFF against multiple state-of-the-art methods across diverse datasets (in vitro mixes, pseudo-bulk, real patient cohorts), convincingly demonstrating superior or competitive performance.

3. Cross-disease validation – The inclusion of COVID-19/nasopharynx data effectively highlights generalizability beyond cancer.

4. Clinical relevance – Application to the beatAML cohort yields biologically interpretable predictions (e.g., LSC-targeting compounds), with substantial literature concordance (72.7%).

5. Transparent and reproducible – Code and data are publicly available with detailed documentation.

Weaknesses:

1. Clarity of the Core Innovation: While the performance is strong, the manuscript could more clearly articulate what specific component of CLIMB’s “iterative, cell-resolved framework” is primarily responsible for its robustness to cross-platform noise compared to other non-DL methods. Is it the iterative reweighting, the cell-level resolution, or a combination? A more focused ablation study would strengthen the claims.

2. Abstract clarity: The abstract could better highlight the methodological advance (cell-level drug sensitivity deconvolution) before diving into results. Suggest to restructure as: “We introduce CLIMB/CLIFF, a framework that… We first validate… We then apply… Our approach…”

3. Technical accessibility: Some methodological descriptions (e.g., EM algorithm, sigmoid linking expression to drug sensitivity) remain dense. Please add a brief conceptual summary in the main text (or a schematic box) explaining how CLIFF’s probabilistic model works intuitively.

4. Figure and supplementary referencing: Some references to supplementary figures are incomplete or misnumbered (e.g., “SFig.??” on p. 72). Occasional formatting inconsistencies and some references are truncated or incomplete (e.g., p. 71, reference to “BisqueRNA [40]” but reference list shows [36]).

5. The discussion of limitations is appropriate but could be expanded regarding potential biases from scRNA-seq reference selection or platform effects. Briefly note how reference quality/coverage might affect CLIMB’s performance in non-hematopoietic contexts.

Reviewer #2: This paper proposes a two-step framework, CLIMB–CLIFF, to infer cell-subtype composition, expression, and drug sensitivity from bulk RNA-seq and bulk drug response data by leveraging reference scRNA-seq. CLIMB performs bulk-to-single-cell deconvolution at the single-cell level, correcting reference-induced bias via empirical Bayes sub-sampling, while CLIFF models bulk drug survival as a mixture of latent subtype-specific survival processes and infers cell-type–specific drug sensitivities through an EM framework. The work addresses a relevant and challenging problem—recovering cell-type–resolved drug response from bulk assays—and provides a probabilistic formulation that explicitly models latent cellular heterogeneity rather than relying on direct regression. Here are my comments:

1. The inference of cell-subtype–specific drug sensitivities relies on variation in cell-subtype proportions across samples. When subtype proportions are highly correlated or show limited variability, the mixture model may suffer from identifiability issues, potentially leading to unstable or non-unique estimates of subtype-specific sensitivities. An explicit discussion of identifiability conditions or sensitivity analyses under correlated subtype proportions would further strengthen the framework.

2. The EM-based optimization involves multiple layers of latent variables and nonlinear parameterization. It is unclear how sensitive the results are to initialization of π or β, or whether multiple local optima exist. The manuscript would benefit from a more thorough analysis of EM convergence properties, including sensitivity to initialization and potential local optima.

3. How is the performance of CLIMB–CLIFF on large datasets? It's better to provide computational complexity or runtime benchmarks.

4. Please clarify how uncertainty in cell-type proportion estimates from CLIMB propagates into the inference of cell-type–specific drug sensitivities in CLIFF.

Reviewer #3: I thank the authors for the opportunity to review their work. They have addressed all comments and I recommend publication.

**********

**Have the authors made all data and (if applicable) computational code underlying the findings in their manuscript fully available?**

The PLOS Data policy requires authors to make all data and code underlying the findings described in their manuscript fully available without restriction, with rare exception (please refer to the Data Availability Statement in the manuscript PDF file). The data and code should be provided as part of the manuscript or its supporting information, or deposited to a public repository. For example, in addition to summary statistics, the data points behind means, medians and variance measures should be available. If there are restrictions on publicly sharing data or code —e.g. participant privacy or use of data from a third party—those must be specified.

Reviewer #1: Yes

Reviewer #2: None

Reviewer #3: Yes

**********

PLOS authors have the option to publish the peer review history of their article (what does this mean?). If published, this will include your full peer review and any attached files.

If you choose “no”, your identity will remain anonymous but your review may still be made public.

**Do you want your identity to be public for this peer review?**  For information about this choice, including consent withdrawal, please see our Privacy Policy.

Reviewer #1: No

Reviewer #2: No

Reviewer #3: No

**Figure resubmission:**

After uploading your figures to PLOS’s NAAS tool - https://ngplosjournals.pagemajik.ai/artanalysis, NAAS will process the files provided and display the results in the "Uploaded Files" section of the page as the processing is complete. If the uploaded figures meet our requirements (or NAAS is able to fix the files to meet our requirements), the figure will be marked as "fixed" above. If NAAS is unable to fix the files, a red "failed" label will appear above. When NAAS has confirmed that the figure files meet our requirements, please download the file via the download option, and include these NAAS processed figure files when submitting your revised manuscript.

**Reproducibility:**

To enhance the reproducibility of your results, we recommend that authors of applicable studies deposit laboratory protocols in protocols.io, where a protocol can be assigned its own identifier (DOI) such that it can be cited independently in the future. Additionally, PLOS ONE offers an option to publish peer-reviewed clinical study protocols. Read more information on sharing protocols at https://plos.org/protocols?utm_medium=editorial-email&utm_source=authorletters&utm_campaign=protocols

# Author comment: Author response to Decision Letter 2

**DOI:** 10.1371/journal.pcbi.1014862.r004

30 Aug 2026

Attachment Submitted filename: ResponseToReviewers_2nd.pdf

# Peer review: Decision Letter 2

**DOI:** 10.1371/journal.pcbi.1014862.r005

**Authors:** Ilya Ioshikhes, Zhaoyuan Fang

28 Sep 2026

Dear Dr. Coudray,

Thanks so much for your careful revision and sticking to scientific rigor. We are pleased to inform you that your manuscript 'Computational deconvolution of drug sensitivity via single-cell–to–bulk transcriptome mapping' has been provisionally accepted for publication in PLOS Computational Biology.

Before your manuscript can be formally accepted you will need to complete some formatting changes, which you will receive in a follow up email. A member of our team will be in touch with a set of requests.

Please note that your manuscript will not be scheduled for publication until you have made the required changes, so a swift response is appreciated.

IMPORTANT: The editorial review process is now complete. PLOS will only permit corrections to spelling, formatting or significant scientific errors from this point onwards. Requests for major changes, or any which affect the scientific understanding of your work, will cause delays to the publication date of your manuscript.

Should you, your institution's press office or the journal office choose to press release your paper, you will automatically be opted out of early publication. We ask that you notify us now if you or your institution is planning to press release the article. All press must be co-ordinated with PLOS.

Thank you again for supporting Open Access publishing; we are looking forward to publishing your work in PLOS Computational Biology.

Best regards,

Zhaoyuan Fang

Academic Editor

PLOS Computational Biology

Ferhat Ay

Section Editor

PLOS Computational Biology

***********************************************************

Reviewer's Responses to Questions

**Comments to the Authors:**

**Please note here if the review is uploaded as an attachment.**

Reviewer #2: All my concerns have been addressed in the revised MS.

**********

**Have the authors made all data and (if applicable) computational code underlying the findings in their manuscript fully available?**

The PLOS Data policy requires authors to make all data and code underlying the findings described in their manuscript fully available without restriction, with rare exception (please refer to the Data Availability Statement in the manuscript PDF file). The data and code should be provided as part of the manuscript or its supporting information, or deposited to a public repository. For example, in addition to summary statistics, the data points behind means, medians and variance measures should be available. If there are restrictions on publicly sharing data or code —e.g. participant privacy or use of data from a third party—those must be specified.

Reviewer #2: None

**********

PLOS authors have the option to publish the peer review history of their article (what does this mean?). If published, this will include your full peer review and any attached files.

If you choose “no”, your identity will remain anonymous but your review may still be made public.

**Do you want your identity to be public for this peer review?**  For information about this choice, including consent withdrawal, please see our Privacy Policy.

Reviewer #2: No

# Editor report: Acceptance letter

**DOI:** 10.1371/journal.pcbi.1014862.r006

**Authors:** Ilya Ioshikhes, Zhaoyuan Fang

PCOMPBIOL-D-25-01865R2

Computational deconvolution of drug sensitivity via single-cell–to–bulk transcriptome mapping

Dear Dr Coudray,

I am pleased to inform you that your manuscript has been formally accepted for publication in PLOS Computational Biology. Your manuscript is now with our production department and you will be notified of the publication date in due course.

The corresponding author will soon be receiving a typeset proof for review, to ensure errors have not been introduced during production. Please review the PDF proof of your manuscript carefully, as this is the last chance to correct any errors. Please note that major changes, or those which affect the scientific understanding of the work, will likely cause delays to the publication date of your manuscript.

Soon after your final files are uploaded, unless you have opted out, the early version of your manuscript will be published online. The date of the early version will be your article's publication date. The final article will be published to the same URL, and all versions of the paper will be accessible to readers.

For Research, Software, and Methods articles, you will receive an invoice from PLOS for your publication fee after your manuscript has reached the completed accept phase. If you receive an email requesting payment before acceptance or for any other service, this may be a phishing scheme. Learn how to identify phishing emails and protect your accounts at https://explore.plos.org/phishing.

Thank you again for supporting PLOS Computational Biology and open-access publishing. We are looking forward to publishing your work!

With kind regards,

Sharmila Kamatchi

PLOS Computational Biology | Carlyle House, Carlyle Road, Cambridge CB4 3DN | United Kingdom ploscompbiol@plos.org | Phone +44 (0) 1223-442824 | ploscompbiol.org | @PLOSCompBiol
